# Silencing the Odorant Binding Protein *RferOBP1768* Reduces the Strong Preference of Palm Weevil for the Major Aggregation Pheromone Compound Ferrugineol

**DOI:** 10.3389/fphys.2018.00252

**Published:** 2018-03-21

**Authors:** Binu Antony, Jibin Johny, Saleh A. Aldosari

**Affiliations:** Chair of Date Palm Research, Department of Plant Protection, College of Food and Agricultural Sciences, King Saud University, Riyadh, Saudi Arabia

**Keywords:** red palm weevil, pheromone-binding protein, aggregation pheromone, RNAi, EAG, olfactometer

## Abstract

In insects, perception of the environment—food, mates, and prey—is mainly guided by chemical signals. The dynamic process of signal perception involves transport to odorant receptors (ORs) by soluble secretory proteins, odorant binding proteins (OBPs), which form the first stage in the process of olfactory recognition and are analogous to lipocalin family proteins in vertebrates. Although OBPs involved in the transport of pheromones to ORs have been functionally identified in insects, there is to date no report for Coleoptera. Furthermore, there is a lack of information on olfactory perception and the molecular mechanism by which OBPs participate in the transport of aggregation pheromones. We focus on the red palm weevil (RPW) *Rhynchophorus ferrugineus*, the most devastating quarantine pest of palm trees worldwide. In this work, we constructed libraries of all OBPs and selected antenna-specific and highly expressed OBPs for silencing through RNA interference. Aggregation pheromone compounds, 4-methyl-5-nonanol (ferrugineol) and 4-methyl-5-nonanone (ferruginone), and a kairomone, ethyl acetate, were then sequentially presented to individual RPWs. The results showed that antenna-specific *RferOBP1768* aids in the capture and transport of ferrugineol to ORs. Silencing of *RferOBP1768*, which is responsible for pheromone binding, significantly disrupted pheromone communication. Study of odorant perception in palm weevil is important because the availability of literature regarding the nature and role of olfactory signaling in this insect may reveal likely candidates representative of animal olfaction and, more generally, of molecular recognition. Knowledge of OBPs recognizing the specific pheromone ferrugineol will allow for designing biosensors for the detection of this key compound in weevil monitoring in date palm fields.

## Introduction

Perception of odorants and chemical sensing are essential processes for the survival of all animals. Research of olfaction and the olfactory system has experienced a quantum leap in recent decades mainly because of patented applications in fields such as biosensors, behavior-based robots, perfumes, and the chemical industry (Du et al., 2013; Yeon et al., 2015; Brito et al., 2016 Ando and Kanzaki, 2017; Garm et al., 2017; Hadagali and Suan, 2017; Leal, 2017; Lutz et al., 2017). Some aspects of human olfaction are difficult to study; conversely, such systems are more readily investigated in insects, organisms that rely strongly on olfaction. Although some differences between olfaction in mammals and insects exist, they are similar in many important ways. In this study, we examined the olfactory system of the red palm weevil (RPW) *Rhynchophorus ferrugineus*, the most invasive and globally important quarantine pest of palm trees. *R. ferrugineus* was introduced to Saudi Arabia from Southeast Asia during the 1980s; it subsequently spread to all Middle Eastern countries and has since migrated into Spain and Southern France (Barranco et al., 1996; Martín et al., 2000; Dembilio and Jaques, 2015; Al-Dosary et al., 2016). The regional and global spread of palm weevil was primarily facilitated by humans *via* the transport of infested offshoots and young or mature date palm trees from weevil-outbreak areas into uninfected areas (Faleiro, 2006; Al-Dosary et al., 2016). When RPWs attack a palm tree, the male weevils release an aggregation pheromone (4-methyl-5-nonanol and 4-methyl-5-nonanone); other RPWs within the vicinity are attracted to the signal, which often leads to a coordinated mass attack and eventually results in the death of the palm tree (Soroker et al., 2005; Faleiro, 2006). Palm weevil aggregation pheromones function in various processes, including defense against predators, overcoming host resistance by mass attack and mate selection. Because of the economic and ecological impacts of this pest, we selected it for study to obtain more extensive knowledge regarding its olfactory communication.

Insect pheromone reception is a complex process in which odorants reach the aqueous environment of the sensillar lymph through multiple pores present on the surface of sensilla. Pheromone-binding proteins (PBPs), odorant receptors (ORs), ionotropic receptors (IRs), sensory neuron membrane proteins (SNMPs), chemosensory proteins (CSPs), and odorant-degrading enzymes (ODEs) are the main proteins of the peripheral olfactory system involved in odorant perception (Hansson and Stensmyr, 2011; Leal, 2013; Zhang et al., 2014; Andersson et al., 2015). The different olfactory protein families involved in insect olfaction have been identified (Hansson and Stensmyr, 2011; Vosshall and Hansson, 2011; Leal, 2013; Missbach et al., 2014; Fleischer et al., 2018), and we selected one set of these genes that code for proteins involved in the first stage of the olfactory process: odorant-binding proteins (OBPs). OBPs interact with particular molecules in the chemical cues of individual odorants and transport them to receptors (Vogt and Riddiford, 1981; Pelosi and Maida, 1995; Pelosi et al., 2006, 2014). Insect OBPs comprise approximately 130–140 amino acids, are abundantly distributed in chemosensilla, consist of four to six α-helical domains and are characterized by four to six conserved cysteines paired into two to three interlocked disulfide bridges (Angeli et al., 1999; Leal et al., 1999; Sandler et al., 2000; Tegoni et al., 2004; Vieira and Rozas, 2011; Pelosi et al., 2014). OBPs are present at high concentrations in the lymph between the dendritic membrane and the cuticular wall (Pelosi et al., 2006, 2014).

Although pheromone detection involving PBPs in insects has been extensively studied, most of the research to date has been performed in moths, mosquitoes and *Drosophila*, whereas there are only a few reports for Coleoptera (Brito et al., 2016; Pelosi et al., 2017). Furthermore, there is a lack of information on olfactory perception and the molecular mechanism by which OBPs participate in the transport of aggregation pheromones in Coleoptera. We selected palm weevil because it is a global pest of palm trees that mainly uses aggregation pheromones to coordinate mass attacks on palm trees, with both host searching and reproductive activity relying strongly on male-produced pheromones. We aimed to identify and characterize a specific subclass of pheromone-specific OBPs by selectively silencing key OBPs using RNA interference and assessing changes in weevil behavior using behavioral trials and electrophysiological recordings. As *R. ferrugineus* is among the world's most invasive pest species of palm trees and this pest has wreaked havoc in the date palm industry in Middle Eastern countries, our current research findings on *R. ferrugineus* OBPs may be applicable in the development of biosensors for pheromone-based monitoring or might be used to screen behaviorally active compounds (attractants or repellents) in an approach similar to “reverse chemical ecology” (Leal et al., 2008; Leal, 2017).

## Materials and methods

### Insect collection and rearing

RPW collections were performed with the direct consent of a cooperating land owner [Saudi Arabia, Al-Kharj region (24.1500°N, 47.3000°E)] in the year 2009. The collected RPWs were maintained in our laboratory on sugarcane stems at 28–30°C with a photoperiod of 18 h:6 h (light: dark), as described previously (Antony et al., 2016, 2017).

### Identification and phylogenetic analysis of RferOBPs

Red palm weevil antennal transcriptome data (Antony et al., 2016) were screened and annotated for candidate OBP genes. Both Blast2GO and manual annotations were performed for the nomenclature, and for convenience, we added a prefix, Rfer (*R. ferrugineus*) for OBP transcripts, followed by the identification number. Reads per kilobase per million (RPKM) values were calculated according to a published formula (Mortazavi et al., 2008). The identified candidates were further annotated and checked for duplications and open reading frame (ORF) identification using the NCBI BLASTx homology search and ORF Finder (https://www.ncbi.nlm.nih.gov/orffinder/). The ORF amino acid sequences were used for phylogenetic tree construction along with selected OBP protein sequences retrieved from NCBI and Protein Data Bank. Multiple sequence alignment was performed using MUSCLE (Edgar, 2004), and a neighbor-joining (NJ) analysis based phylogenetic tree was reconstructed using MEGA v6 (Kumar et al., 2016), with the tree branches supported by 1,000 bootstrap replications.

### Selection of candidate OBPs for gene silencing

#### Tissue-specific expression analysis

For tissue-specificity and qRT-PCR studies, the antennae, snout, legs, thorax, abdomen, and wings were excised from 20-day-old adult insects. Total RNA was extracted from 30 mg of tissue for each sample using PureLink RNA Mini Kit (Ambion, USA), and first-strand cDNA was synthesized using SuperScript IV Reverse Transcriptase (Invitrogen, Carlsbad, CA, USA) according to the manufacturer's instructions. The quality and quantity of the RNA and cDNA were examined using a NanoDrop spectrophotometer (Thermo, Delaware, USA). Primers were designed using Primer3 software (Untergasser et al., 2012) with the following parameters: Tm, 56–60°C; GC content, 40–50%; and product size, 190–200 bp (Table S1). Touchdown polymerase chain reaction (PCR) [95°C for 5 min, 35 cycles of 95°C for 1 min, 60°C (touchdown to 54°C) for 30 s and 72°C for 30 s; and one cycle at 72°C for 10 min] was carried out using GoTaq Green PCR Master Mix (Promega, USA), and the PCR products were evaluated by 2.5% agarose gel electrophoresis alongside a 100-bp DNA ladder (Solis BioDyne, Tartu, Estonia) as a marker and visualized using ethidium bromide (Promega, USA) staining.

#### Relative expression analysis by qRT-PCR

cDNAs were prepared from RNA extracted from the antennae of 20-day-old insects, as mentioned above. qRT-PCR was carried out using SYBR Green PCR Master Mix (Life Technologies, USA) with three biological and three technical replicates according to the manufacturer's instructions. The oligonucleotide primers used were the same as those used in the tissue-specific studies, and tubulin (Table S1) was employed to normalize gene expression. The relative RferOBP expression levels were measured by the 2^−ΔΔC^_T_ method (Schmittgen and Livak, 2008). The following thermal programme was used to perform the PCR amplification: holding stage at 50°C; 95°C for 2 or 5 min; 40 cycles of 95°C for 15 s; and 60°C for 32 s; and a continuous melting curve stage of 95°C for 15 s, 60°C for 1 min, 95°C for 30 s, and 60°C for 15 s. The qRT-PCR products were examined by 3% agarose gel electrophoresis and visualized *via* ethidium bromide staining.

#### Rapid amplification of cDNA ends (RACE) and generation of the full-length sequence

The SMARTer rapid amplification of cDNA ends technique (SMARTer RACE Kit, Clontech, CA, USA) was used to obtain the full-length sequences of candidate OBPs by amplifying both cDNA ends (5′ and 3′ ends). The 5′ and 3′ RACE cDNAs were prepared from total RNA of adult *R. ferrugineus* antennae, as described (Soffan et al., 2016). Gene-specific primers (GSPs) for 5′- and 3′-RACE were designed based on partial *RferOBP23, RferOBP107, RferOBP1768*, and *RferOBPu1* nucleotide sequences (Table S1). The amplification reactions were carried out as follows: 95°C for 5 min; 30 cycles of 95°C for 1 min, 65°C (touchdown to 60°C) for 30 s and 72°C for 2 min; and one cycle at 72°C for 10 min. The amplified PCR products were purified using Wizard SV Gel Purification Kit (Promega, USA) and cloned into the pGEM-T vector (Promega, USA) followed by transformation into JM109 competent cells (Promega, USA). The plasmids were isolated from bacteria, sequenced in both directions (ABI 3500, Life Technologies, MD, USA), aligned and annotated using a BLASTx homology search.

#### Structural and functional analyses

Amino acid similarity and identity were calculated using the SIAS tool (http://imed.med.ucm.es/Tools/sias.html). Sequence logos of the aligned *R. ferrugineus* OBP orthologs were created using WebLogo 3.1 (Crooks et al., 2004). The DISULFIND web server tool (http://disulfind.dsi.unifi.it) was used to predict the distribution of disulfide bonds. Compute pI/Mw (http://web.expasy.org/compute_pi/) was used to predict the theoretical pI (isoelectric point) and Mw (molecular weight). The SignalP 4.0 Server program (http://www.cbs.dtu.dk/services/SignalP) and Euk-mPLoc 2.0 (http://www.csbio.sjtu.edu.cn/bioinf/euk-multi-2/) were applied to predict signal peptides and subcellular localization, respectively. The 3DLigandSite tool (http://www.sbg.bio.ic.ac.uk/3dligandsite) was utilized to predict ligand-binding sites in the proteins. The Phyre2 tool (http://www.sbg.bio.ic.ac.uk/$\sim$phyre2/) was employed to predict secondary structures, and PyMol (https://pymol.org/2/) was used to visualize simulated three-dimensional structures.

#### RferOBP silencing by RNA interference (RNAi)

We used plasmids containing the full-length OBP ORF as template DNA to synthesize double-stranded RNA (dsRNA). ORF reverse primers with a T7 overhang and T7 forward primers (Table S1) were used to amplify and linearize ORFs, which were rechecked by direct sequencing (ABI 3500, Life Technologies, USA). dsRNA synthesis was performed using MEGAscript RNAi Kit (Life Technologies, USA) according to the manufacturer's instructions, and the results were quantified using a NanoDrop 2,000 (Thermo Scientific, DE, USA). dsRNA was examined by 1% agarose gel electrophoresis to evaluate the integrity and efficiency of duplex formation. We selected 10-day-old *R. ferrugineus* pupae for RNAi experiments, and 40 ng/μL dsRNA (in 20 μL) was injected at a depth of 0.5 cm into the first dorsal segment of the abdomen, close to the thorax, using a 0.5-mL BD Micro-FineTM PLUS syringe (Becton, Dickinson Co., NJ, USA). dsRNA-injected RPW pupae were maintained as previously described (Soffan et al., 2016). As two separate controls, RPW pupae were injected with nuclease-free water (hereafter referred to as “NFW”) or not injected (hereafter referred to as “NI”). The adults emerging at 21 days were further subjected to quantification of gene silencing (qRT-PCR), behavioral assays using an olfactometer, and electrophysiological recording using an electroantennogram (EAG), as described below.

#### Gene silencing validation by qRT-PCR

cDNAs were prepared from RNA extracted from the antennae of each individual insect in the experimental (dsRNA injected) and control (NFW and NI) groups and used as template for qRT-PCR. Reactions were carried out using SYBR Green PCR Master Mix (Life Technologies, USA) according to the manufacturer's instructions, with six biological and six technical replicates. *Tubulin* and β*-actin* primers were used to normalize gene expression (Table S1). The relative expression levels of OBPs in the silenced vs. control groups were measured by the 2^−ΔΔC^_T_ method (Schmittgen and Livak, 2008). PCR amplification, data analysis, statistical analysis, and gel evaluation were performed as described above.

### Behavioral and electrophysiological assays

#### Olfactometer assay

The olfactometer assay was used to evaluate the responses to stimuli by the dsRNA-injected, NI and NFW groups of RPW adults. We used a customized olfactometer unit (Volatile Collection System Co, Gainesville, FL) consisting of a Y-tube (main-tube length: 47 cm; arm length: 68 cm; diameter: 5 cm; with 40-cm-long/2-cm-diameter plastic tubes in each arm connected to the source of the stimulus), an air-delivery system (humidified air and carbon filter), and a stimulus container (diameter: 8 cm, length: 10 cm). A commercial aggregation pheromone contained 4-methyl-5-nonanol (ferrugineol) and 4-methyl-5-nonanone (ferruginone) at the approximate ratio of 9:1 (ChemTica Int., Costa Rica) and ethyl acetate (Sigma Aldrich, USA) were used in one arm of the instrument, and charcoal-filtered air was applied in the other arm. We used ethyl acetate because several studies have reported that it enhances the efficacy of weevil catch (Soroker et al., 2005; Shagagh et al., 2008; Al-Saoud, 2013; Vacas et al., 2013, 2017). The unit was operated at a pressure of 15 psi and a zero air inlet flow of 1.2 L per minute. Adult insects were starved overnight, and the response to stimuli was recorded three times for each insect. Failure to move within 5 min in the olfactometer Y-tube was classified as “no response.” As our preliminary study showed that NI and NFW adult RPWs exhibit similar responses to the stimulus, further assays were carried out with the NI and dsRNA-injected groups only; each group comprised 16 adult RPWs of similar age (ratio of 1:1, male: female). The numbers of times (three times on different experimental days: *n*) each RPW chose “air,” “stimulus,” or “no response” were recorded, and the results are expressed as percentages of the total.

#### Electroantennography (EAG)

To validate the effect of gene silencing using RNAi, insects with positive results in the olfactometer assay were subjected to electroantennography. Six adult RPWs were tested per group (dsRNA injected, NFW injected and NI) at the age of 21 days. After demobilization using CO_2_ for 1–2 min, the antennae of each insect were excised from the base. Each antenna was then attached to the electrode holders of an EAG system (Syntech, Hilversum, Netherlands) using SPECTRA 360 electrode gel (Parker Lab, Inc. Fairfield, NJ, USA) and subjected to a constant flow of humidified air. Each insect from the experimental groups was exposed to three different stimuli, (4RS,5RS)-4-methylnonan-5-ol, (Phe1) (>92% purity, ChemTica Int., Costa Rica), 4(RS)-methylnonan-5-one (Phe2) (>92% purity, ChemTica Int., Costa Rica), and ethyl acetate (Sigma Aldrich, USA), at concentrations of 0.02 mg/mL (diluted in *n*-hexane).

A glass Pasteur pipette with a filter paper strip inside (with 4 μL of the stimulus compound) was used to deliver the stimulus *via* an air-stimulus controller (Model CS-55 Ver.2.7, Syntech, Hilversum, The Netherlands) fitted with a charcoal filter. Odor stimulation puffs were applied twice at 0.1-s intervals and with 20–30-s intervals between each odor compound. The antennal response to each stimulus was recorded using a Syntech Acquisition IDAC-2 controller connected to a computer and processed using GC-EAD 2012 v1.2.4 (Syntech, Kirchzarten, Germany).

#### *RferOBP1768* expression analysis in male and female *R. ferrugineus*

Differences in *RferOBP1768* expression in adult male and female *R. ferrugineus* weevils were compared by qRT-PCR. Antennae from 21-day-old male and female adult insects were excised, and total RNA extraction and cDNA synthesis were performed as described above. Three biological and three technical replicates were used for male and female RPWs; *RferOBP1768* expression was normalized to that of *tubulin* and β*-actin* (Table S1) and calculated using the 2^−ΔΔC^_T_ method (Schmittgen and Livak, 2008).

#### Statistical analysis

The mean fold change, 2^−ΔΔC^_T_ values (Livak and Schmittgen, 2001), were calculated using MS Excel (Microsoft corporation, USA). Three experimental groups, consisting of dsRNA RferOBP-injected (dsRNA), not-injected (NI), and NFW-injected groups, were established with triplicate biological and technical replicates. Significant differences among the experimental groups for qRT-PCR, the olfactometer assay and EAG were assessed using one-way analysis of variance (ANOVA), followed by multiple-comparison testing with the least significant difference (LSD) test (*P* < 0.05) (for the olfactometer assay and qRT-PCR) or with Tukey's HSD test (for EAG analysis) (Stelinski and Tiwari, 2013) using SPSS program v24. Homogeneous subsets in both the olfactometer and EAG assays were identified by Waller-Duncan statistics (α = 0.05) using SPSS program v24 (IBM SPSS statistics, NY, USA).

## Results

### Identification and selection of candidate OBPs for gene silencing

**Figure 1:**
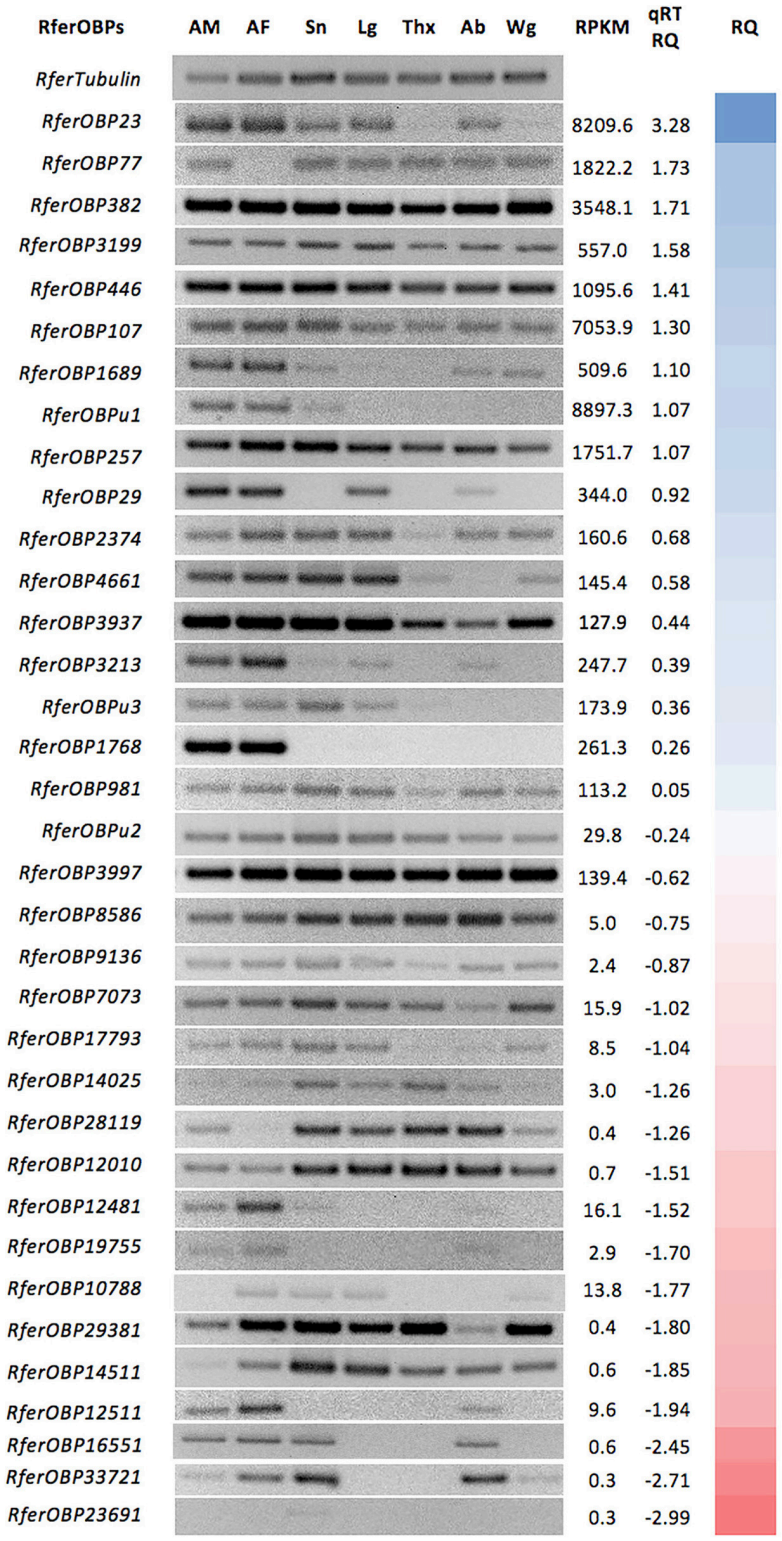
Relative tissue-specific expression analysis of 36 OBPs identified from *Rhynchophorus ferrugineus*. Tissues used are indicated as AM (male antennae), AF (female antennae), Sn (male snout), Lg (male legs), Thx (male thorax), Ab (male abdomen), and Wg (male wings). *tubulin* was used to normalize gene expression. Expression of all *RferOBPs* in the antenna was quantified by qRT-PCR, and the mean fold changes in gene expression compared to *tubulin* are provided under qRT-RQ. The color gradient indicates the relative level of expression from higher (blue) to lower (red). Primer details and PCR product sizes are provided in Table S1. The original gel image (with DNA ladder) is provided in Figure S1.

A comprehensive search of the RPW antennal transcriptome identified 38 OBPs, and we confirmed these transcripts by checking for duplication based on BLASTx hits and concluded that 36 OBPs are present in RPW (Table S2). Sequence homology and characterization of the RPW OBPs were performed. RPKM values calculated for the assembled OBP transcripts are presented in Figure 1; this analysis revealed highly abundant transcripts of three OBPs (*RferOBPu1, RferOBP23*, and *RferOBP107*) in the RPW antennal transcriptome (RPKM > 7,000).

### Tissue-specific expression analysis demonstrates antenna-specific RferOBPs

We aimed to investigate OBP(s) involved in the first stage of detecting and transporting aggregation pheromones of *R. ferrugineus*. For an initial clue regarding their function, we first mapped expression of 36 OBPs in the antennae and other body parts of *R. ferrugineus*. Among the 36 OBPs, only one candidate OBP (*RferOBP1768*) was found to be exclusively antenna specific (Figure 1). *RferOBPu1* exhibited antenna-enriched expression but low expression in the snout (Figure 1). Similarly, *RferOBP3213* and *RferOBP29* showed antenna-enriched expression but with low expression in the leg and abdomen (Figure 1). We identified four candidates with reduced expression in the antennae than in other body parts (*RferOBP8586, RferOBP7073, RferOBP12010*, and *RferOBP14511*), and the remaining OBPs displayed ubiquitous expression patterns (Figure 1). Among the highly expressed candidate OBPs, *RferOBP23* was expressed in all tissues studied except the thorax and wings, whereas *RferOBP107* was ubiquitously expressed in all tissues (Figure 1). Interestingly, two OBPs (*RferOBP77* and *RferOBP28119*) exhibited no expression in the antenna of females but were expressed in all other tissues. For antenna-enriched OBPs, expression of *RferOBP12511* and *RferOBP12481* was high in RPW females compared to males, with low expression in the snout and abdomen (Figure 1).

### Relative expression analysis reveals key OBPs in the *R. ferrugineus* antenna

Expression of all OBPs in the *R. ferrugineus* antenna was quantitatively measured, and the RQ values are provided in Figure 1. Based on qRT-PCR data, *RferOBP23, RferOBP77, RferOBP382, RferOBP3199*, and *RferOBP446* are the highly expressed OBPs in *R. ferrugineus*. Compared to other OBPs, *RferOBPu23* and *RferOBP107* were found to be highly expressed in the antenna (Figure 1). Other candidate genes showing high expression in the antenna were *RferOBP77, RferOBP382, RferOBP3199*, and *RferOBP446*. Conversely, *RferOBPu1* expression was lower than that of the highly expressed OBPs (Figure 1). The antenna-specific candidate gene *RferOBP1768* also displayed moderate expression in the antenna (mean 0.76-fold change normalized by tubulin gene expression), as shown in Figure 1. The antenna-enriched OBPs *RferOBP12511, RferOBP19755*, and *RferOBP12481* all showed very low expression (Figure 1).

### Structural and functional analyses

#### Molecular cloning, full-length sequencing, and phylogenetic analysis

**Figure 2:**
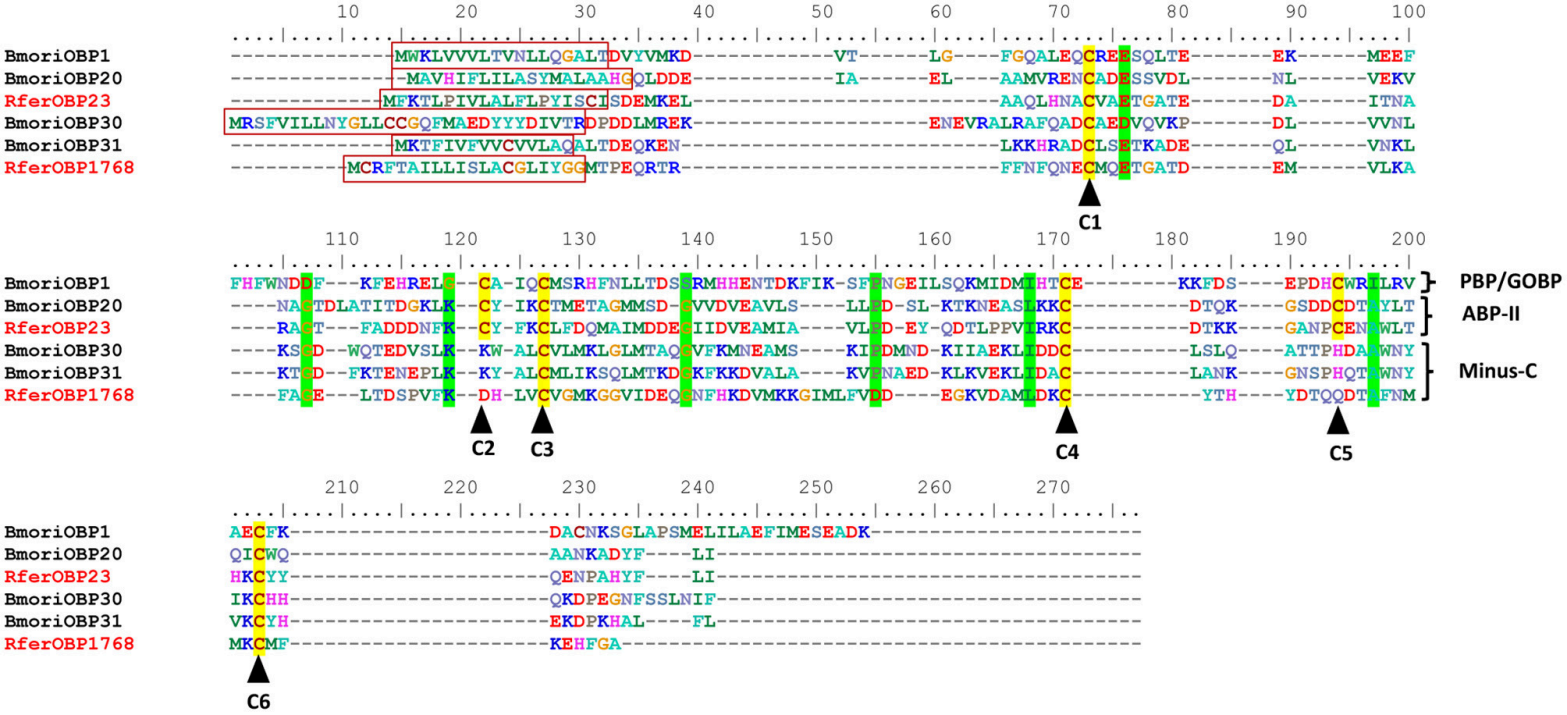
Sequence alignment of *Rhynchophorus ferrugineus* odorant binding proteins *RferOBP1768* and *RferOBP23, Bombyx mori* (Bmori) PBP1, PBP20, PBP30, and PBP31. Highly conserved cysteine residues are marked by dark arrowheads. Signal peptides are boxed. Residues highlighted in bright-green have high (>90%) consensus values. Conserved residues are shown with a green background. Because the four OBPs (*B. mori)* are from different insect orders, homologies are low. Sequence logos of the aligned *R. ferrugineus RferOBP1768* and *RferOBP23* orthologs are shown in Figure S3.

We selected *RferOBP23, RferOBP107, RferOBP1768*, and *RferOBPu1* for full-length cloning and analysis because the first two were found to be highly expressed and the last two were found to be antenna specific and antenna enriched, respectively. Full-length OBP sequences were obtained for *RferOBPu1, RferOBP23, RferOBP107*, and *RferOBP1768* using the SMARTer RACE technique, assisted by a primer walking sequencing strategy. The *RferOBPu1, RferOBP23, RferOBP107*, and *RferOBP1768* genes were confirmed to have full lengths of 612, 643, 703, and 636 bp, respectively, with ORFs of 396, 402, 429, and 399 bp, corresponding to 131, 133, 142, and 132 amino acids (Figure 2). The theoretical pI (isoelectric point)/Mw (molecular weight) of the proteins encoded by *RferOBPu1, RferOBP23, RferOBP107*, and *RferOBP1768* are 4.41/15.16, 4.44/14.98, 4.72/15.88, and 5.08/14.92, respectively. We identified OBP extracellular localization, a typical characteristic of OBP proteins, using the Euk-mPLoc 2.0 server. The full-length amino acid sequences of *RferOBPu1* shows 25.11, 16.66, and 48.09% identity with *RferOBP23, RferOBP107*, and *RferOBP1768*, respectively. *RferOBP23* exhibits 25 and 32.33% identity with *RferOBP107* and *RferOBP1768*, respectively, and *RferOBP107* exhibits 16.66% identity with *RferOBP1768*. Using SignalP-4.1 euk predictions, we identified a highly divergent signal peptide at the N-terminal region, as shown in Figure 2.

A NJ rooted tree of various different annotated OBPs and *Bombyx mori* OBPs (Gong et al., 2009) was used as a reference to classify RferOBPs. We focused on *RferOBP23, RferOBP107, RferOBP1768*, and *RferOBPu1* based on the results obtained in tissue-specificity studies and relative OBP expression analysis. We identified *RferOBP23* and *RferOBP107* as belonging to ABP II subfamilies, and *RferOBP1768* and *RferOBPu1* were classified as Minus-C subfamilies (Figure S2). The Minus-C *RferOBP1768* clade also includes other *R. ferrugineus* OBPs, namely, *RferOBP12511, RferOBPu1, RferOBP1689*, and *RferOBP19755. RferOBP1768* shows 65.1 and 66.9% amino acid identity with *RferOBP1689* and *RferOBP19755* (Figure S3). In our tree, the *RferOBP1768* clade, with 91% bootstrap support, forms a clade with *TcasOBPC06* and *TcasOBPC09* (Figure S2). The tree also revealed that *RferOBP23* belongs to an orthologous sequence group containing *TcasOBP6, TcasOBP8*, and *BmoriOBP20*, with 56, 54, and 44% bootstrap support, respectively (Figure S2), and that *RferOBP107* belongs to an orthologous sequence group containing *BmoriOBP21*, with 51% bootstrap support (Figure S2). The *RferOBP23* clade contains *RferOBP3213* and *RferOBP107, BmoriOBP20* and OBPs from scarab beetles (*Anomala osakana, AosaOBP; Anomala octiescostata, AoctOBP; Anomala cuprea, AcupOBP*) and the Japanese beetle *Popillia japonica* (*PjapOBP*) (Wojtasek et al., 1998; Nikonov et al., 2002).

**Figure 3:**
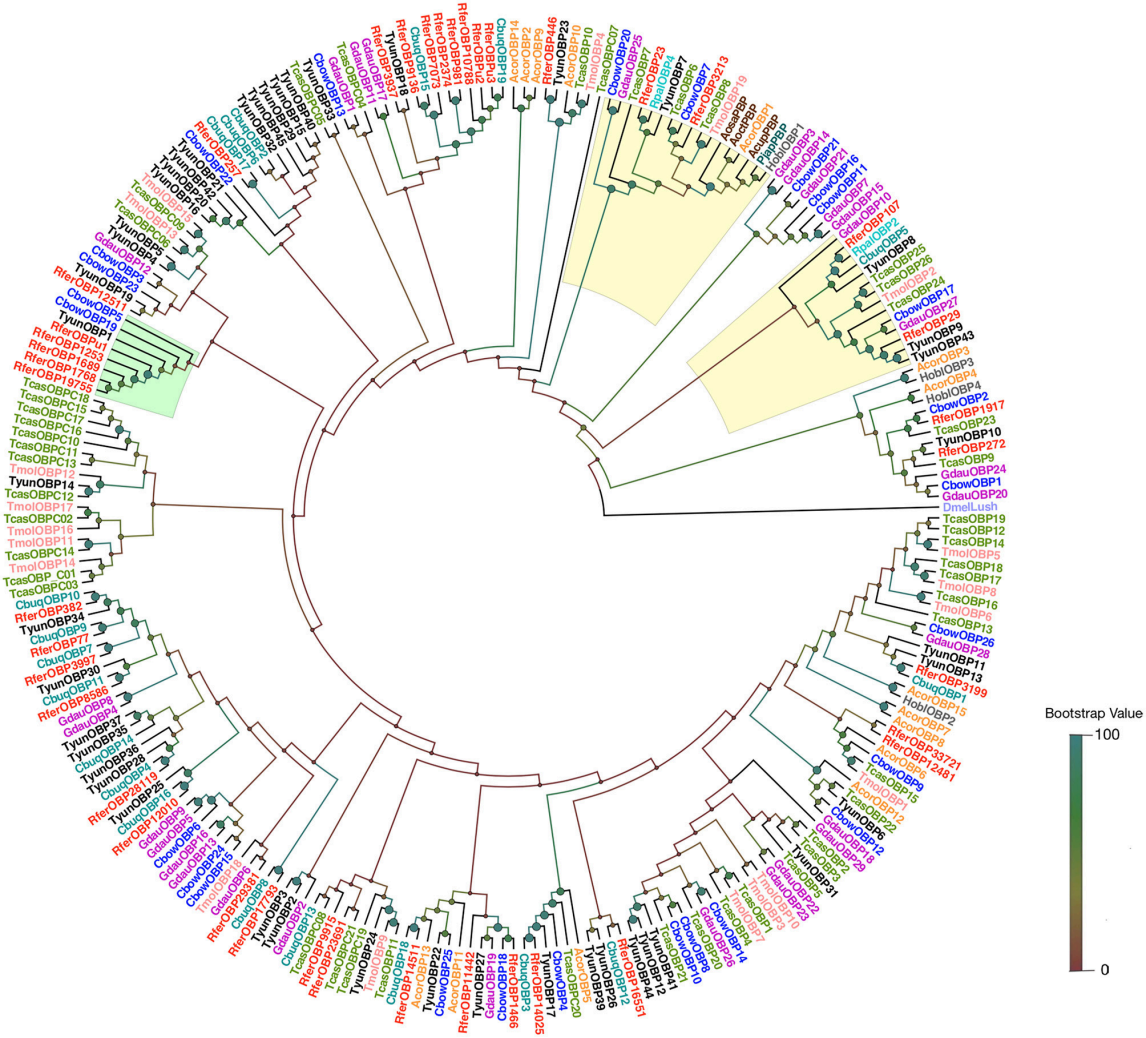
A neighbor-joining (NJ) rooted tree of OBPs from coleoptera. OBP amino acid sequences of *R. ferrugineus* and *R. palmarum; RpalOBP2* and *RpalOBP4* (Nagnan-Le Meillour et al., 2004) and 10 other coleopterans [*T. yunnanensis* (Liu et al., 2018); *H. oblita* (Li K. et al., 2017); *C. buqueti* (Yang et al., 2017a) *C. bowringi* (Li X. et al., 2015); *G. daurica* (Li K. et al., 2017); *T. molitor* (Liu et al., 2015); *T. castaneum* (Dippel et al., 2014), *A. corpulenta* (Li X. et al., 2015)], scarab beetles and the Japanese beetle (Wojtasek et al., 1998; Nikonov et al., 2002) were retrieved from the GenBank. The NJ analysis was computed using MEGA (v.6.0) [statistical method: NJ; phylogeny test: bootstrap method; model: JTT model and gaps/missing data treatment: pairwise deletion] and generated with a bootstrap procedure using 1,000 replications and the bootstrap values are indicated at the nodes. The branch containing *Drosophila* OBP LUSH (*DmelLush* PDB: 2GTE) was used as an outgroup to root the tree. The *RferOBP1768* and *RferOBP23* (ABP II) clades are highlighted in green and yellow, respectively. The OBPs from different species were marked with different colors. Phylogenetic tree was visualized with the software FigTree (http://tree.bio.ed.ac.uk/software/figtree/) and branch appearance was colored based on the bootstrap values. Scale: 0.4 amino acid substitutions per site.

OBP NJ tree was constructed based on amino acid sequences using *R. ferrugineus* and *Rhynchophorus palmarum; RpalOBP2* and *RpalOBP4* (Nagnan-Le Meillour et al., 2004) and 10 other coleopterans [*Tomicus yunnanensis* (Liu et al., 2018); *Holotrichia oblita* (Li K. et al., 2017); *Cyrtotrachelus buqueti* (Yang et al., 2017a) *Colaphellus bowringi* (Li X. et al., 2015); *Galeruca daurica* (Li K. et al., 2017); *Tenebrio molitor* (Liu et al., 2015); *Tribolium castaneum* (Dippel et al., 2014), *Anomala corpulenta* (Li X. et al., 2015)], scarab beetles, and the Japanese beetle (Wojtasek et al., 1998; Nikonov et al., 2002) (Figure 3). Members of the ABP II clade show diversity in sequence and function. Gene expansion was identified within this clade, particularly in the cluster of *RferOBP23* and *RferOBP107* (Figure 3). The phylogenetic tree shows that *RferOBP23* is similar to the American palm weevil (APW), *R. palmarum* OBP4 (*RpalOBP4*) (Nagnan-Le Meillour et al., 2004), with sound bootstrap support (86%); and also found related to *T. yunnanensis, TyunOBP7; T. castaneum, TcasOBP7; RferOBP3213, T. molitor, TmolOBP19; TcasOBP8, H. oblita, HoblOBP1*, and OBPs from scarab beetles and the Japanese beetle (Figure 3). Similarly, the phylogenetic analysis identified ortholog of *RferOBP1768* from other coleopteran insects, which include *TyunOBP1* and *C. bowringi; CbowOBP19* and *CbowOBP5* (Figure 3).

#### RNAi-based gene silencing of RferOBPs

**Figure 4:**
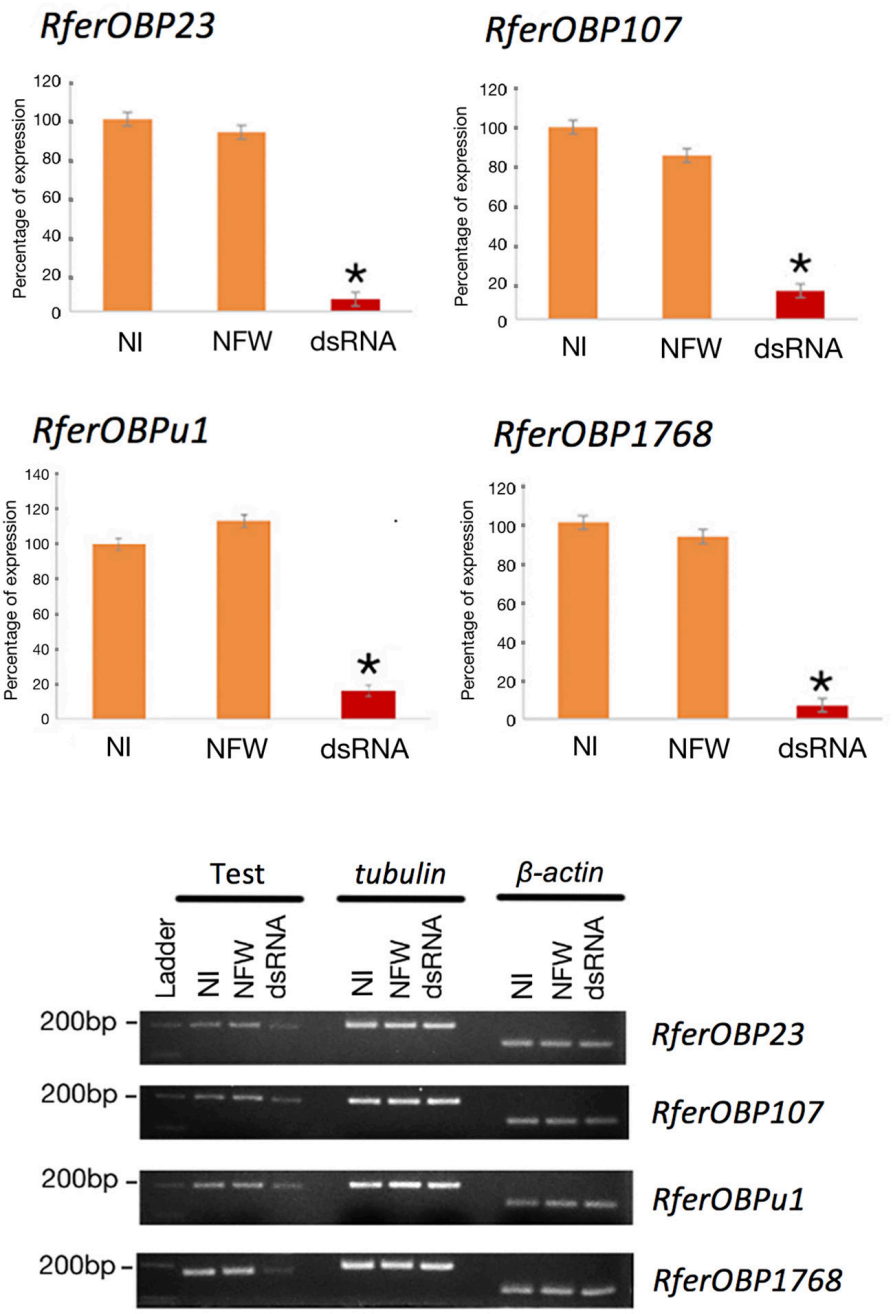
RNAi-based gene silencing of candidate *RferOBPs* validated by qRT-PCR. Error bars represent the SEM. Significance was measured using one-way ANOVA with respect to the control. ^*^A significant reduction in gene expression (*P* < 0.001). Representative visual bands of 1. NI (control), 2. NFW, and 3. dsRNA-injected groups. The first row shows expression of RferOBPs in the different experimental groups, and the second and third rows show *tubulin* and β-*actin* expression (refer Table S1) in the different experimental groups.

We selected *RferOBP23, RferOBP107, RferOBPu1*, and *RferOBP1768* for the RNAi experiments because the first two OBPs were highly expressed and the remaining two were antenna enriched and antenna specific, respectively. Regarding *RferOBP1768* silencing, qRT-PCR gene expression data with normalization using multiple control genes (*tubulin* and β*-actin*) showed 99.44 and 92.77% silencing (in 21-day-old adult weevils) compared to NFW and NI RPWs, respectively (Figure 4). For *RferOBP107, RferOBP23*, and *RferOBPu1*, we achieved 85.52, 93.48, and 85.21% silencing, respectively, compared to the NI samples (Figure 4, *P* < 0.001), and we achieved 73.29, 98.25, and 85.44% silencing for *RferOBP107, RferOBP23* and *RferOBPu1*, respectively, compared to the NFW experimental group (Figure 4, *P* < 0.001).

### Behavioral and electrophysiological assays

#### Olfactometer assay

**Figure 5:**
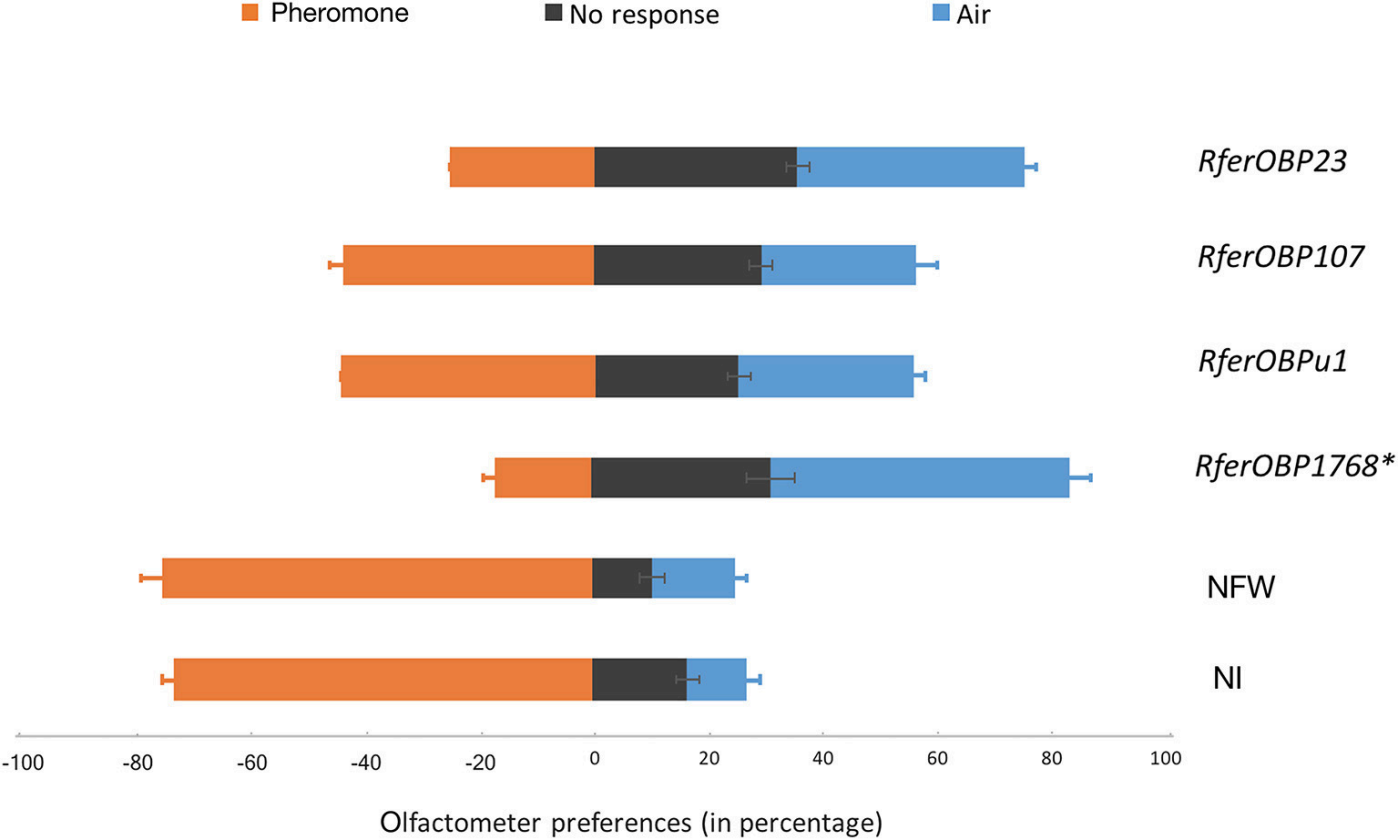
Olfactometer preferences exhibited by OBP-silenced (dsRNA-injected) RPWs against NI (not-injected) and NFW-injected insects to pheromone (4-methyl-5-nonanol (ferrugineol) and 4-methyl-5-nonanone (ferruginone) at the approximate ratio of 9:1), air or no response, as expressed as a percentage of the total (*n* = 12–16). Error bars represent the SEM. ^*^The most highly significant difference in response (*P* < 0.001) compared to the control. The detailed statistical analysis is provided in Table S3.

The silencing of *RferOBP1768* and *RferOBP23* also resulted in behavioral changes in *R. ferrugineus* in response to commercial aggregation pheromone in the olfactometer assay. Among *RferOBP1768*-silenced insects, 31% showed no response, 17% recognized the pheromone, and the remaining 52% moved away from the pheromone, toward the filtered-air arm of the Y-tube olfactometer (*F* = 43.8, *df* = 2, and *P* = 0.0002). In insects with *RferOBP23* silencing, a similar pattern of olfactometer response was observed, but with only 40% moving toward the air; 35% showed no response, and 25% responded to the pheromone (*F* = 19.5, *df* = 2 and *P* = 0.002). ANOVA of the percentages of OBP-silenced RPW adults that moved away from the pheromone compared with the control indicated significantly more efficient *RferOBP1768* silencing compared to the other RferOBPs (Figure 5). The olfactometer assay response was calculated for each group tested, and the results are presented as a percentage of the total number of insects in Table 1. Only 17% of *RferOBP1768*-silenced RPWs were able to detect the aggregation pheromone, which was significantly different from the results for all other experimental groups (*F* = 81.27; *df* = 4; *P* < 0.0001) (Table 1, Table S3). Nevertheless, 52% of *RferOBP1768*-silenced RPWs moved away from the pheromone, which was also significantly different from all other experimental groups (*F* = 9.66; *df* = 4; *P* < 0.0001) (Table 1). In the case of *RferOBP23*-silenced insects, only 25% responded to the commercial aggregation pheromone, also a significant reduction compared to the control (Figure 5, Table S3). In contrast, more than 40% of *RferOBPu1*- and *RferOBP107*-silenced RPW adults responded to the aggregation pheromone. We selected *RferOBP1768-, RferOBPu1-, RferOBP107*-, and *RferOBP23*-silenced RPW adults for EAG studies.

**Table 1:** Olfactometer preferences of not-injected, NFW-injected and dsRNA-injected insects toward pheromone, air and no response, as expressed as percentages of the total.

**Treatment groups**	***N***	**Response toward pheromone (%)**	**No response (%)**	**Response toward air (%)**
NI	16	72.92 (2.08)^d^	16.67 (2.08)^a,b^	10.42 (2.08)^a^
NFW	16	75.00 (3.61)^d^	10.42 (4.17)^a^	14.58 (2.08)^a^
RferOBP23	16	25.00 (0.00)^b^	35.42 (2.08)^d^	39.58 (2.08)^c^
RferOBP107	16	43.75 (2.08)^c^	27.08 (3.61)^c,d^	29.17 (2.08)^b^
RferOBPu1	12	44.44 (2.08)^c^	25.00 (3.61)^b,c^	30.56 (2.08)^b^
RferOBP1768	16	16.67 (2.08)^a^	31.25 (2.08)^c,d^	52.08 (2.08)^d^
*F*-value		81.275	33.921	9.668
*P*-value		<0.0001	0.001	<0.0001

*The SEM is provided in parentheses. Significance was measured by using one-way ANOVA followed by LSD analysis, with a significance level of P < 0.05. Homogeneous subsets were identified by Waller-Duncan statistics (α = 0.05), and the results are represented as a, b, c, and d*.

#### Electroantennography (EAG)

**Figure 6:**
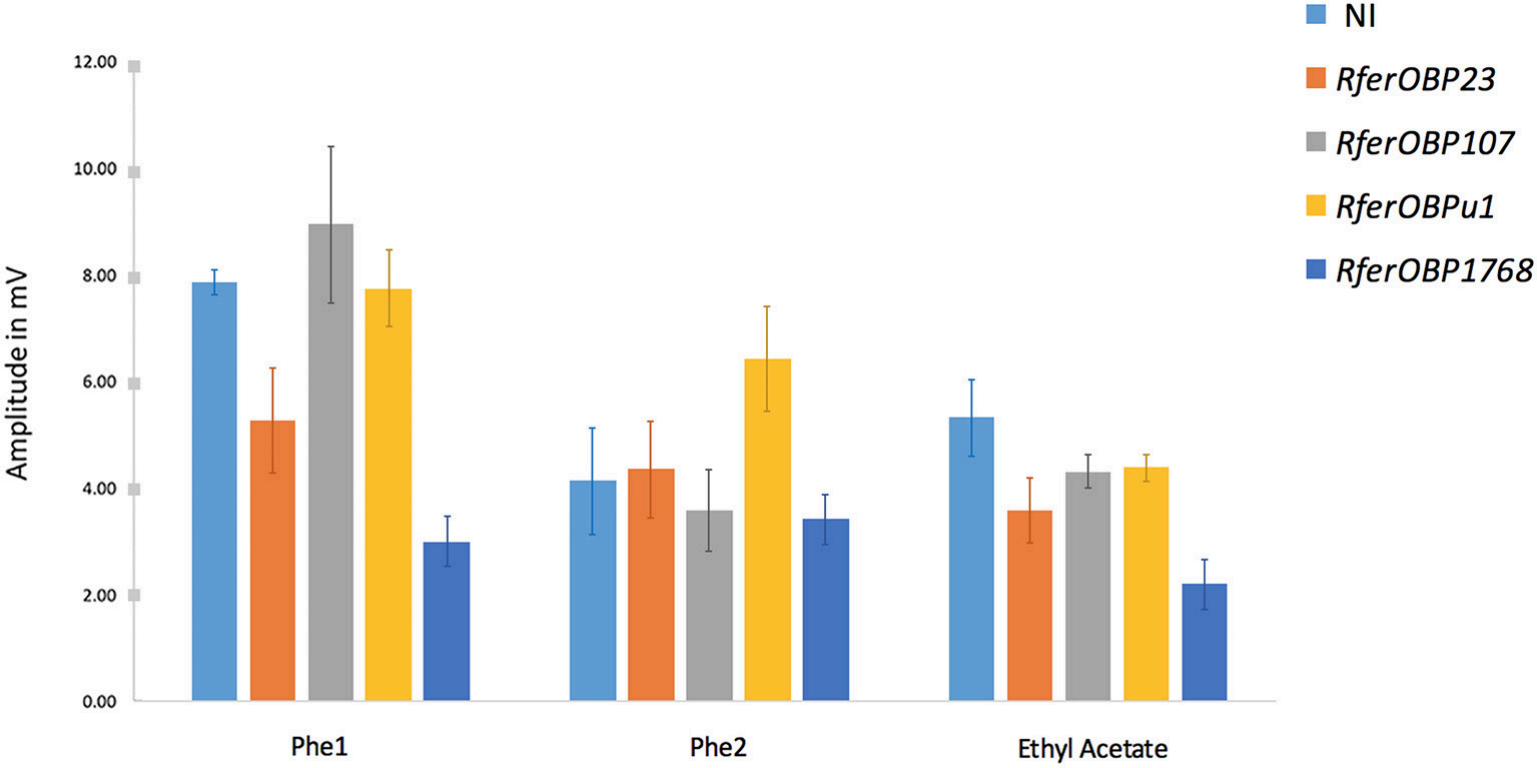
Comparison of EAG responses from treated and non-treated samples to three different stimuli, as represented as three groups. The values represent the amplitudes of signals in mV, and the error bars represent the SEM. The detailed statistical analysis is provided in Table S4.

To validate the altered behavior observed for *RferOBP* dsRNA-injected *R. ferrugineus* adults in the olfactometer assay, RPW antennae were excised and exposed to three stimuli (Phe1, Phe2, and EA) in EAG analysis. The antennal response to the different stimuli for each experimental group was recorded and compared to that of the NI group (Figure 6). *RferOBP1768*-silenced RPWs showed significantly reduced responses to Phe1 compared to NI control RPWs (*F* = 7.52; *df* = 4, *P* = 0.005) (Figure 6, Table S4). We also noted that *RferOBP1768*-silenced insects exhibited a comparatively attenuated response to ethyl acetate than the respective controls and other experimental groups (*F* = 4.45; *df* = 4, *P* = 0.025) (Figure 6, Table S4). Nevertheless, the response of *RferOBP1768*-silenced insects to Phe1 was significantly lower than that of *RferOBP23-, RefOBP107*-, and *RferOBPu1*-silenced RPW adults, whereas *RferOBP107* and *RferOBPu1* responded to Phe1 normally (Table S4). Moreover, all RPW experimental groups responded normally to Phe2 (Figure 6).

Table S4 presents the data of a comparison of RPW responses to Phe1, Phe2, and ethyl acetate among the different experimental groups and the NI control. In *RferOBP1768*-silenced insects, the difference in response to Phe1 was significant, with a *P*-value of 0.015. Interestingly, we observed a moderate reduction in the response to ethyl acetate in *RferOBP1768*-silenced RPWs compared to the control (*P* = 0.015). In contrast, *RferOBP23-, RferOBP107*-, and *RferOBPu1*-silenced RPWs responded to ethyl acetate normally (Figure 6, Table S5). It is worth mentioning that a moderate difference in response to Phe1 was observed for *RferOBP23*-silenced RPWs; however, based on Tukey's HSD, it was insignificant compared to the response of *RferOBP1768*-silenced RPWs to Phe1 (Figure 6, Table S5).

#### Structure modeling of *RferOBP1768* and *RferOBP23*

**Figure 7:**
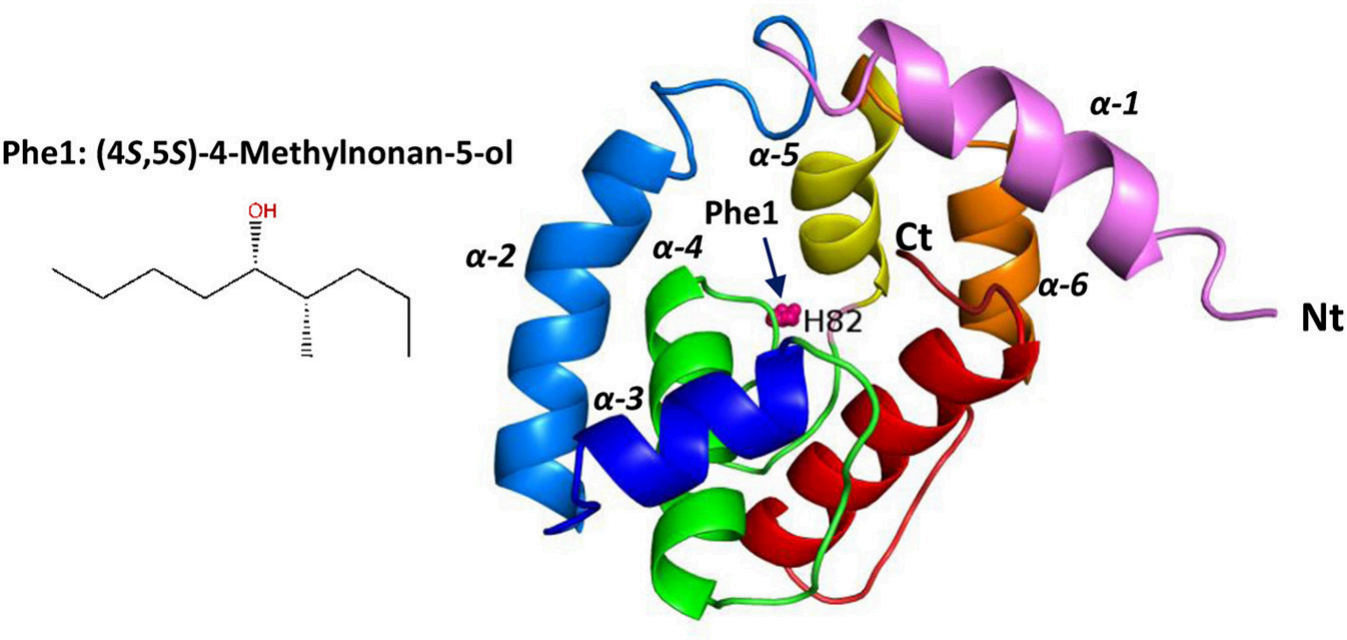
Three-dimensional structures, shown in ribbon representation, of *RferOBP1768* prepared based on the highly ranked structural homolog *Locusta migratoria* OBP1 (PDB 4pt1) using the PHYRE tool. The structures were visualized using PYMOL *v*2.0.4. The rainbow coloring mode is applied to the Cα ribbons: the N-terminus (Nt) is blue, and the C-terminus (Ct) is red. The ligand [Phe1: (4RS,5RS)-4-methylnonan-5-ol] is represented as spheres. The six helices (α1–α6) are indicated. The predicted binding site of *RferOBP1768* corresponds to H82, which is likely involved in pheromone binding. Phe1: chemical structure of the major pheromone compound (4*S*,5*S*)-4-methylnonan-5-ol (source: Pherobase).

Based on behavioral trials and electrophysiological recordings, we selected *RferOBP1768* for further study and built a structure model using the PYRE 2 web model (Kelley et al., 2015) based on the crystal structure (86% of residues modeled at >90% confidence) of *Locusta migratoria* odorant binding protein 2 (Zheng et al., 2015)(PDB: 4PT1). As we noted a moderate affinity of *RferOBP23* for Phe1, we also created a model based on insect pheromone/odorant binding proteins (OBPs) (88% of residues modeled at > 90% confidence) (Lartigue et al., 2004) (PDB: 3BJH). The modeled *RferOBP1768* 3-D structure is typical of insect OBPs, comprising six α-helices folded into a very compact and stable globular structure. The predicted binding site of *RferOBP1768* corresponds to H82, which is likely involved in pheromone binding (Figure 7). The *RferOBP1768* protein contains 6 cysteine residues, which can form three disulfide bonds [(C2, C124), (C14, C67), and (C36, C107)]. The predicted binding site of *RferOBP23* corresponds to Ile79 and Asp80, which are likely involved in pheromone binding (Figure S4). The *RferOBP23* protein contains 6 cysteine residues, which can form three disulfide bonds [(C18, C34), (C61, C65), and (C103, C112)] (Figure S3).

#### *RferOBP1768* expression analysis in male and female *R. ferrugineus*

The relative expression of *RferOBP1768* was low in *R. ferrugineus* males compared to that in females (expression was normalized using multiple house-keeping genes: *tubulin* and β*-actin*). We observed a slight difference in expression patterns between males and females, with mean fold change values of 0.0472 and 0.069, respectively, for male and females (**Figure 9**). However, the values were not significantly different (*P* = 0.764) and thus did not define a sex-specific variation in *RferOBP1768* expression, which supported our tissue-specific expression analysis (Figure 1).

## Discussion

As a first step to understanding the function of the large repertoire of OBPs involved in pheromone communication in the highly invasive quarantine pest *R. ferrugineus*, we first identified antenna-specific *RferOBP1768*. We then demonstrated that dsRNA injection caused a significant reduction in the electrophysiological recording of the response to a major aggregation pheromone compound, (4RS,5RS)-4-methylnonan-5-ol (ferrugineol), leading to altered behavior that ultimately resulted in the failure to sense the pheromone in a behavioral assay. The results of the behavioral assay regarding the response to ferrugineol supported the physiological role of *RferOBP1768* as the ferrugineol-binding protein that aids in the capture and transport of aggregation pheromones to receptors in the palm weevil *R. ferrugineus*. In contrast, no significant differences in electrophysiological response to ferrugineol and a minor pheromone compound, 4-methyl-5-nonanone (ferruginone), or to a kairomone, ethyl acetate, were reported for other highly expressed orthologous OBPs. With 92–94% OBP silencing achieved with dsRNA-injected *R. ferrugineus*, our study demonstrates that pheromone communication disruption can occur through *RferOBP1768* silencing. Our study results have an application in the field of OBP-based biosensors, and *RferOBP1768* is the most promising candidate for fabricating biosensors to detect ferrugineol in “reverse chemical ecology” approaches (Leal et al., 2008; Leal, 2017). RNAi and electrophysiological approaches are widely used and well-accepted methods for the characterization of OBPs, especially PBPs, in insects (Xu et al., 2005; Laughlin et al., 2008; Biessmann et al., 2010; Pelletier et al., 2010). In addition, the use of RNAi and electrophysiological approaches in characterizing OBPs is well documented in mosquitoes (Biessmann et al., 2010; Pelletier et al., 2010), *Drosophila* (Xu et al., 2005; Laughlin et al., 2008); *Aphis gossypii* (Rebijith et al., 2016); *Adelphocoris lineolatus* (Zhang et al., 2017); and *Helicoverpa armigera* (Dong et al., 2017). Such attempts have confirmed the role of OBPs in olfaction, as carriers of hydrophobic odorants and pheromones through the aqueous environment of the sensillum lymph to ORs (Leal, 2013). Regardless, no RNAi studies to date related to the role of OBPs have been reported in beetles, though several studies have been performed to characterize odorant co-receptors by RNAi and electrophysiological approaches (Soffan et al., 2016; Zhang et al., 2016). To the best of our knowledge, the current study is the first attempt to specifically characterize aggregation pheromone-specific OBPs using a gene silencing approach in a beetle.

We previously identified 38 OBPs and grouped *R. ferrugineus* OBPs into different OBP-subfamilies (Antony et al., 2016) to provide a basis for evolutionary and functional analyses of OBPs in palm weevil. In previous degenerate PCR approaches, two OBPs were identified from a species related to RPW, the APW *R. palmarum* (Nagnan-Le Meillour et al., 2004), and more recently, tissue-specific expression profiling was reported for 11 OBPs from *R. ferrugineus* (Yan et al., 2016). OBPs have been reported from a wide range of insect species, and the number of OBPs in some species with sequenced genomes ranges from a 12 in ant species, at least 35 putative OBPs in *Drosophila*, and 44 in silkworms to more than 100 in certain mosquitoes (Hekmat-Scafe et al., 2002; Gong et al., 2009; Smith et al., 2011; Vieira and Rozas, 2011; Manoharan et al., 2013). We used qRT-PCR and tissue-specific expression patterns to select candidate OBPs for RNAi. The tissue-specific expression analysis revealed only one antenna-specific OBP gene, *RferOBP1768*, with all other OBPs showing expression in other specific tissues or in all tissues. The fundamental role of OBPs in olfaction is supported by several studies demonstrating that OBPs involved in pheromone transport are specifically expressed in the antenna (Shanbhag et al., 2001; Pelosi et al., 2006). This approach has been applied to several lepidopteran insects to identify the PBPs that are uniquely expressed in antennae (Vogt and Riddiford, 1981; Vogt et al., 1991; Nikonov et al., 2002; Pelosi et al., 2006; Zhou, 2010; Sun et al., 2013; Jiao et al., 2016).

**Figure 8:**
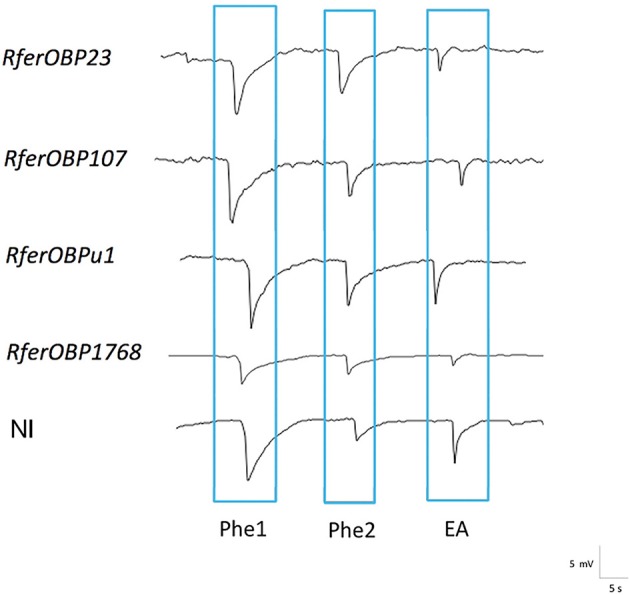
EAG responses from NI control weevils and those silenced for *RferOBPs* represented as waveforms. Measurements were performed at 10 mV and 15-s intervals. 4-Methyl-5-nonanol was Phe1, and 4-methyl-5-nanone was Phe2. Both pheromone compounds were dissolved in hexane. EA indicates ethyl acetate (kairomone).

Expression of OBPs in different tissues may be related to their roles in other physiological functions in that tissue; however, it has also been proposed that the type of sensillum where an OBP is expressed, rather than the organ, might define the role of the protein in taste or olfaction (Pelosi et al., 2006; Zhou, 2010). We also selected two highly expressed OBPs for characterization because the majority of insect OBPs studied to date are highly expressed in chemosensory structures, including antennae (Brito et al., 2016), and we thus initially presumed that these OBPs might be involved in pheromone detection in *R. ferrugineus*. We did not select the second-most highly expressed *R. ferrugineus* OBP, *RferOBP77*, because this candidate was found not to be expressed in the female antenna (Figure 1) and an earlier report indicated that male *R. ferrugineus*-produced aggregation pheromone can attract both female and male RPWs (Hallett et al., 1993; Oehlschlager et al., 1995). Nevertheless, preliminary studies on the scarab beetle *A. octiescostata* showed expression of PBPs in both sexes (Nikonov et al., 2002). Phylogenetic analysis of *R. ferrugineus* OBPs revealed that the *RferOBP1768* clade also contains other *R. ferrugineus* OBPs, such as *RferOBP19755, RferOBP12511*, and *RferOBP1689*; however, we did not select these candidates for further study because the first two showed very low expression and the last showed ubiquitous expression (Figure 1). Although we ranked *RferOBP1689* as the seventh-most highly expressed candidate, we eliminated it from further study because we observed expression in wings (Figure 1). We included *RferOBPu1* in silencing experiments due to its antenna-enriched expression and because this candidate shows high identity to *RferOBP1768* (48% amino acid sequence identity and 99% bootstrap support), though the predicted protein structures and binding sites (H82 for *RferOBP1768* and H81 for *RferOBPu1*) are surprisingly similar. Regardless, our results showed that *RferOBPu1*-silenced RPWs respond to Phe1, Phe2, and EA normally (Figure 8). Based on the observed sequence identity and similar binding sites, we assume that *RferOBP1768* would function as a ferrugineol-specific OBP and be able to activate pheromone-sensitive neurons, whereas *RferOBPu1* would act as an antagonist-binding protein and be able to activate different neurons or bind to non-pheromone ligands for other functions.

**Figure 9:**
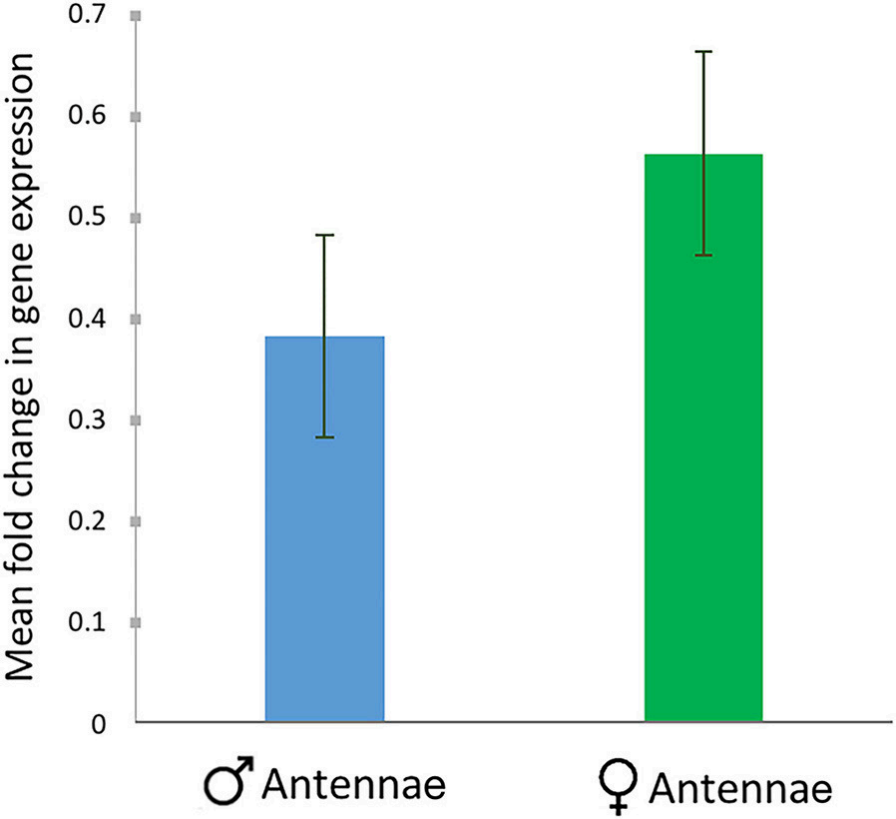
Comparison of *RferOBP1768* expression in the antenna of male and female RPWs. The mean fold change in gene expression compared to multiple endogenous controls (*tubulin* and β-*actin*) is provided along with the SEM as error bars. No significant difference was observed between male and female expression patterns, as based on one-way ANOVA (*P* = 0.764, *F* = 7.708).

The olfactometer assay showed significantly altered behavior in *RferOBP1768*-silenced *R. ferrugineus*, and EAG recordings indicated that *RferOBP1768* silencing in palm weevils decreases the insect's strong preference for the aggregation pheromone ferrugineol. We observed a perfect correlation between the reduction in *RferOBP1768* transcript levels and modest antennal responses to the pheromone, and the simplest explanation is that *RferOBP1768* may be involved in the detection of ferrugineol. Moreover, we observed slight differences in expression of *RferOBP1768* in both sexes (Figure 9), and the higher expression level in *R. ferrugineus* females indicates different roles in pheromone perception for males and females. A similar observation of differential expression patterns of key OBPs in male and female insects has been reported previously (Maida et al., 2005; Campanini et al., 2017). The differential detection of ferrugineol in males and females associated with distinct sexual behaviors might be because more *RferOBP1768* is required in females, leading to differentiation in expression level. Another possibility is that females may be able to recognize ferrugineol as a species-specific pheromone to elicit important ecological and behavioral consequences, and hence a different form of olfactory perception occurs in female *R. ferrugineus*. There is no female-produced sex pheromone reported thus far in *R. ferrugineus*, and studies have shown that male-produced aggregation pheromone can attract females to the vicinity and ultimately facilitate mating (Hallett et al., 1993; Oehlschlager et al., 1995; Kaakeh, 1998; Abdel-Azim et al., 2012; Inghilesi et al., 2015).

The results of our study also indicate that *RferOBP23* can detect the *R. ferrugineus* aggregation pheromone; however, based on EAG recordings, this result was not significant (*P* = 0.153) compared to that of *RferOBP1768*. The results of behavioral trials and EAG clearly proved a significantly higher discriminatory affinity for *RferOBP1768* compared to *RferOBP23* toward ferrugineol (Figure 8, Table S5). However, considering the ability of *RferOBP23* to detect ferrugineol, we assume that this OBP can accommodate ferrugineol in addition to other unknown ligands, which need to be determined. As *RferOBP23* is a highly expressed OBP in *R. ferrugineus*, its broad binding abilities indicate that it may act as a general odorant binding protein (GOBP) to carry out a variety of functions. In addition, both OBPs may be associated with the detection of ferrugineol, as reported in the case of *H. armigera*, in which both *HarmPBP1* and *HarmPBP2* are responsible for the detection of the major sex pheromone component, *Z*11–16:Ald (Dong et al., 2017). Studies have also shown that OBPs undergo specific conformational changes upon binding to their ligand molecules, and only in selected cases do such changes enable the OBP to interact with the OR and generate a physiological response (Laughlin et al., 2008). Thus, GOBPs that do not undergo suitable conformational changes may not be able to trigger the subsequent physiological response. Several previous studies have reported the phenomenon of OBPs exhibiting a broad spectrum of binding (Maida et al., 2000; Plettner et al., 2000; Campanacci et al., 2001; Leal et al., 2005a,b; Zhou, 2010). Regardless, there are studies, mostly in lepidopteran insects, suggesting that PBPs can selectively bind to sex pheromone components produced by females; the pheromone (*E*,Z)-10,12-hexadecadienol (bombykol) is the specific ligand for *B. mori* PBP (*BmorPBP1*), and the pheromone component (*E*,*Z*)-6,11-hexadecadienal is the specific ligand for *Antheraea polyphemus* PBP (*ApolPBP1*) (Sandler et al., 2000). However, studies have also demonstrated that OBPs can also bind to a wide range of odorant chemicals (Honson et al., 2003, 2005; Zhou, 2010; Zhou et al., 2010; Venthur et al., 2014) and that different PBPs can bind to the same sex pheromone component (Campanacci et al., 2001; Guo et al., 2012; Gu et al., 2013). Despite studies to support selective binding, a full understanding of the discriminative ability of OBPs remains elusive (Pelosi et al., 2014, 2017; Brito et al., 2016). Nevertheless, all the OBP functional studies mentioned above are based solely on lepidopteran and dipteran insects (Zhou, 2010; Leal, 2013; Pelosi et al., 2014, 2017; Brito et al., 2016), and hence, the results may not hold in the case of coleopteran insects. In *R. ferrugineus*, the male-produced aggregation pheromone ferrugineol can equally attract both female and male adult weevils (Hallett et al., 1993; Oehlschlager et al., 1995); hence, GOBP/PBP may not be specifically involved in both sexes. Our results are consistent with the idea that *RferOBP1768* is antenna specific, and our phylogenetic analysis and structural analyses classified *RferOBP1768* in the Minus-C category (Hekmat-Scafe et al., 2002; Gong et al., 2009). In addition to ferrugineol, *RferOBP1768* also exhibits affinity toward a kairomone, ethyl acetate, as we observed in the EAG recording of dsRNA-injected weevils (Figure 8). These broad binding abilities indicate that *RferOBP1768* may not act as a GOBP/PBP for specific pheromone binding; however, there is no report on the functional identification of OBPs in coleopteran insects involved in aggregation pheromone detection for comparison with the results in *R. ferrugineus*. Although the *T. castaneum* genome is available and *TcasOBP6* and *TcasOBP9* were found to be similar to *RferOBP1768* (Figure S2), no specific role of OBPs has yet been proposed; thus, it is difficult to suggest a common function of these clustered OBPs in the family. Similarly, *C. bowringi; CbowOBP5* and *CbowOBP19* (Li X. et al., 2015) and *T. yunnanensis*; *TyunOBP1* (Liu et al., 2018) were found to be similar to *RferOBP1768* (Figure 3), no functional role of these OBPs has yet been proposed. However, it is worth noting that the *T. yunnanensis* transcriptome data revealed the presence of 45 OBPs, from which *TyunOBP1* was more antennal-specific and significantly expressed in the antennae (Liu et al., 2018).

In the current study, we focused on *RferOBP23, RferOBP107, RferOBP1768*, and *RferOBPu1* based on results obtained in tissue-specificity studies and relative OBP expression analysis. Phylogenetic analysis revealed that the ABPII subfamily of OBPs containing *RferOBP23, RferOBP107*, and *RferOBP3213* from *R. ferrugineus* form a clade with PBPs from scarab beetle and Japanese beetle (Wojtasek et al., 1998; Nikonov et al., 2002) (Figure 3), with *RferOBP23* and *RferOBP3213* showing more than 50% amino acid identity with PBPs from these beetles (Figure S4). Scarab beetle and Japanese beetle PBPs are reported to be involved in detecting the sex pheromone enantiomers (*S*)-japonilure and (*R*)-japonilure, respectively, based on the single-neuron technique and are the only PBPs identified thus far from Coleoptera (Wojtasek et al., 1998). As per phylogenetic analysis, *RferOBP3213* is a promising candidate for testing in silencing experiments; however, based on its low expression in the snout, leg and abdomen in tissue-specific expression analysis and its low expression (RQ-value 0.39) in qRT-PCR analysis, we did not include further evaluate this candidate. It is interesting to note that the *RferOBP3199*, which is ubiquitously expressed in *R. ferrugineus* (Figure 1); we identified a putative ortholog in another curculionid, *C. buqueti*; *CbuqOBP1* (Yang et al., 2017a) (97% bootstrap support, Figure 3) and this putative PR reported to be related to the recognition of dibutyl phthalate, a sex pheromone analog in *C. buqueti* (Yang et al., 2017b). Based on our phylogenetic analysis, we speculate that in Curculionidae such genes may have the same ancestral gene, and the possibility is that the OBP expansions facilitated the adaptive evolution of a variety of specialized functions among different species.

*Rhynchophorus ferrugineus* has recently received greater attention due to its invasiveness and quarantine pest status. Conventional methods have proven ineffective for the management of palm weevil, leading to proposals of synthetic biology approaches intended at disrupting pheromone communication, given that olfaction interference has the potential to interrupt critical behaviors such as host and mate selection, ultimately disrupting reproductive success and causing weevil population decline (Antony et al., 2016; Soffan et al., 2016). We previously reported *RferOrco* silencing, and together with OBP silencing in *R. ferrugineus via* dsRNA injection, this approach is promising for the disruption of pheromone communication in *R. ferrugineus* (Soffan et al., 2016). To enable use of the RPW RNAi technique, *RferOBP1768* and *RferOrco* dsRNA delivery *via* feeding or effective delivery systems such as synthetic nanoparticle and engineered microorganisms (Baum et al., 2007; Kolliopoulou et al., 2017), the generation of transgenic bacteria that express dsRNA (Tian et al., 2009), the chemical synthesis of siRNA (Gong et al., 2011) or the application of dsRNA in a spray form to facilitate its spread might offer excellent future prospects for controlling this invasive pest. Another promising area is the development of OBP-based biosensors for the detection of odorants. Such a biotechnological application of OBPs against *R. ferrugineus* is yet to be explored, and thus our identification of a ferrugineol-specific OBP from RPW holds great promise for the development of insect behavioral attractants or repellents or artificial biosensors. Considering that pheromone communication is an important aspect of *R. ferrugineus* attack of palm trees, where individual insects use male aggregation pheromone to find trees and coordinate a group attack that eventually leads to palm tree death, understanding the key OBP involved in this mechanism is a significant achievement for the date palm industry. Although substantial antennal transcriptome data are available for coleopteran insects (Dippel et al., 2014; Liu et al., 2015, 2018; Li X. et al., 2015; Li X.M. et al., 2015; Li K. et al., 2017; Li L. et al., 2017), PBPs from scarab beetle and Japanese beetle are the only coleopteran OBPs identified thus far (Wojtasek et al., 1998; Nikonov et al., 2002). However, their functional characterization has not been reported, and hence there is much work needed in exploring the olfactory mechanism in beetles and the pattern of OBP relatedness between beetles. Further aspects of the identified candidate OBPs, such as structure and ligand-binding capability, also need to be explored.

## Data availability statement

All relevant data are within the paper and its Supporting Information files. The OBP nucleotide sequences can be obtained from the Transcriptome Shotgun Assembly project DDBJ/EMBL/GenBank under the accession number GDKA00000000. The OBP contig names are mentioned in abbreviated form; for example, the *RferOBP23* GenBank acc. no. is GDKA01000023, and the *RferOBP1768* GenBank acc. no. is GDKA01001762. The full length sequences reported in this paper have been deposited in the GenBank database (accession nos.: *RferOBP1768*, MH026102; *RferOBPu1*, MH026103; *RferOBP23*, MH026104; *RferOBP107*, MH026105, and *RferOBP3213*, MH026106).

## Author contributions

BA conceived the study and acquired the grant, also participated in its design, coordination and supervision; SA paid the expenses for the weevil collection and culture; JJ and BA carried out the laboratory experiments and analyzed the data; BA wrote the paper with contributions from JJ, and all authors read and approved the final manuscript.

### Conflict of interest statement

The authors declare that the research was conducted in the absence of any commercial or financial relationships that could be construed as a potential conflict of interest.
