# Supplementary material for: Silencing the Odorant Binding Protein RferOBP1768 Reduces the Strong Preference of Palm Weevil for the Major Aggregation Pheromone Compound Ferrugineol
Source: Front Physiol. 2018 Mar 21;9:252. doi: 10.3389/fphys.2018.00252 (PMC5871713; doi:10.3389/fphys.2018.00252)
Supplement: Supplementary file 1 [file Table1.PDF]

Table S1. Primers used for tissue specific expression analysis, qRT-PCR, gene silencing quantification, RACE sequencing and RNAi experiments.

Sequence Name		Primer sequence (5' to 3')	Tm (˚C)	Product size (bp)
RferOBPu1	Forward	TCAAGGAACACTTGGTGTGT	54.3	185
	Reverse	TGTCATTCGGTAAGCCAGTT	54.1	
RferOBPu2	Forward	GGCATTCGCTTTCTGTCTCT	55.2	179
	Reverse	AAGTGTGGAAGGCTGTTTCT	54.4	
RferOBPu3	Forward	CTTCAGTGACGATCCCAAGTT	54.7	188
	Reverse	TTTCTTCGGGAGAGCCTTTG	55	
RferOBP2374	Forward	GCTTCAGCGACGATCCAAA	55.7	195
	Reverse	ATGCCGTTTCTTCAGGAGTG	55.2	
RferOBP257	Forward	TACAAACACTGCGTGGAAGA	54.2	204
	Reverse	CTGGTCCTCGTTTCCTTTGA	54.6	
RferOBP29	Forward	TCGCCAACACCATTGCTAAG	55.8	196
	Reverse	CCGTCCGTTGTCATCCATTT	55.5	
RferOBP3199	Forward	ATGTGCTCAACAGGAGAAGG	54.8	174
	Reverse	TTGGCTCTTTCGATCCAGTC	54.7	
RferOBP3213	Forward	CGTAAAGGCCATGTCTGATGA	54.8	213
	Reverse	ATGGTTGCCTCAACGTCTATT	54.3	
RferOBP7073	Forward	GGCCTTTGCATTCTGCATATC	54.6	205
	Reverse	AGGGTTGGCTTTGTAGTAGC	54.8	
RferOBP8586	Forward	ACATAGGTTGTCAGGGCATC	54.6	191
	Reverse	GACATGACGGATGTGTGTCT	54.6	
RferOBP9136	Forward	AACCGGCATAGATGGAAACA	54.3	224
	Reverse	GAGTTCCCTCCTTTCCAAGAC	55.1	
RferOBP981	Forward	CTTCAGCGACGATCCCAAAT	55.3	186
	Reverse	TCTTCGGGAGTGTCCTTCTT	55.4	
RferOBP9915	Forward	CGCTGATGAAACGAACGTAAA	53.7	213
	Reverse	TCGCAATCGACAGCTACTT	54	
RferOBP77	Forward	GAAACTGAAACGCGGTGAAA	54	183
	Reverse	GAGCAAGCACTGACGATTTG	54.5	
RferOBP382	Forward	TGCTCTGTACGTCGATGATG	54.4	206
	Reverse	CGGTAACCTGTTTCGCTTTG	54.4	
RferOBP446	Forward	ACTTCGTGGACGACAATTCC	55.1	196
	Reverse	AGGTAAACGGTATCGCAAGC	55.2	
RferOBP1768	Forward	CCTCGCTTGCGGACTAATA	54.4	175
	Reverse	ACCAACACAAACCAGATGAT	52.1	
RferOBP3937	Forward	CTGCTAAACCTTTGACAGATG	51.4	199
	Reverse	GGTTGAAAGACGTTGTTGG	51.9	
RferOBP3997	Forward	GTCGAACTACCACCCAACTT	54.6	194
	Reverse	ACGGCAGCATCGTCTTTAT	54.4	
RferOBP4661	Forward	AAGAAGTCATCAACACTTGCC	53.3	172

	Reverse	GCCTCCAATTTGCGATATACA	53	
RferOBP12010	Forward	GCAGCCACTCGCATAGATAAA	55.1	214
	Reverse	CGCACTCCTGCAACAAAGTA	55.6	
RferOBP14025	Forward	CCCGAATGCTCTACGACAAT	54.8	184
	Reverse	TTCTCATCCTCGCCGTTTAC	54.7	
RferOBP23691	Forward	TATCACAACTGCGGATCTGG	54.5	204
	Reverse	GCTTCCTTGTTCAGTGTGTTC	54.2	
RferOBP28119	Forward	CCTGTGTTGCCGCTTTATTG	54.8	198
	Reverse	TGGAGATGCAGAGCGAATG	55	
RferOBP29381	Forward	GCACATGAACAATGCCAGTC	54.8	184
	Reverse	TGACCTGTCCCATCGTATCT	55	
RferOBP33721	Forward	ATGCATGGTTGATTGCCTTT	53.4	187
	Reverse	CACATTGCAACCCTCTGATTC	54.2	
RferOBP10788	Forward	CCTTTATGGGCCTGAGTTTCT	54.7	229
	Reverse	ACGTCAGCTTGTACATCACC	54.9	
RferOBP12481	Forward	TGTGAAGAAAGCTGCTGGTAT	54.3	172
	Reverse	GCATTTGGTCATTTGGTAGGC	54.8	
RferOBP12511	Forward	GTGAGGAATGCTTTCAAGGG	53.8	217
	Reverse	ATGCTGTCTCTTCAGGACTC	53.9	
RferOBP14511	Forward	GAAGCCGAGAATGCTACACA	54.9	192
	Reverse	CACGTCATCCAAACCCTTCT	55	
RferOBP16551	Forward	AAACTCAACTTTCGGAGAGAA	51.7	182
	Reverse	AAGCATATCCTTAACGTGACT	51.4	
RferOBP1689	Forward	TTCAAGGCGAGTGCATGAT	54.7	210
	Reverse	CATCGACTTTCGTTTCATCGC	54.5	
RferOBP17793	Forward	TGCGATATCCGCTCTTACAC	54.5	210
	Reverse	TCGTCGGTTTGTAGGTTTCC	54.8	
RferOBP19755	Forward	TGACGGATTCACCACTGTTC	54.9	191
	Reverse	AGCGGTTTCCTGTTGAGTAT	53.9	
RferOBP23	Forward	CAATGCACGAGCTGGTACATTCG	58.6	168
	Reverse	TTTCTGATTACTGGCGGTAGGGT	57.9	
RferOBP107	Forward	TCGCAGGGCATATCGACATAGA	57.7	155
	Reverse	TTGCAAAGGCTCGGTCACAGT	59.6	
RferTubulin	Forward	GCTACCTTCATCGGCAACTC	55.8	196
	Reverse	CGGTGGCTTCTTGGTATTGT	55.3	
Rferbeta-actin	Forward	AAAGGTTCCGTTGCCCTGAA	57.3	129
	Reverse	TGGCGTACAAGTCCTTCCTG	57	

Sequence Name		Primer sequences (5’ to 3’)	Tm (˚C)
RferOBP23	Forward	ATGTTTAAAACGTTACCGATAG	48.7
	Reverse	TAATACGACTCACTATAGGGTTAAATTAAGAAATAATG	55.4
RferOBP107	Forward	ATGTTCAGTTTAACCGAACTAG	50.4
	Reverse	TAATACGACTCACTATAGGGTCAAAATACATAGTATATATC	56.8
RferOBP1768	Forward	ATGTGTCGTTTTACTGCTATTTTA	50.9
	Reverse	TAATACGACTCACTATAGGGTTAAGCTCCAAAATGTTCTTTAAAC	61.2
RferOBP3213	Forward	ATGAGACAGCATTCGATTTTTTTGGGT	57.7
	Reverse	TAATACGACTCACTATAGGGTTAAATTAACATGTATTCCGCAAAAT	61.1
RferOBPu1	Forward	ATGAATAATTTGGTGGTTTTATTACTT	50.2
	Reverse	TAATACGACTCACTATAGGGTTACAGACCGAAATGTTCATTATACAA	62.1
