# Supplementary material for: Silencing the Odorant Binding Protein RferOBP1768 Reduces the Strong Preference of Palm Weevil for the Major Aggregation Pheromone Compound Ferrugineol
Source: Front Physiol. 2018 Mar 21;9:252. doi: 10.3389/fphys.2018.00252 (PMC5871713; doi:10.3389/fphys.2018.00252)
Supplement: Supplementary file 2 [file Table2.PDF]

Table S2. *R. ferrugineus* OBP new names and the corresponding names from Antony et al, 2016

Antony et al., 2016	RferOBP naming in the current study	Yan et al., 2016 (GenBank acc. nos.)
RPW1_contig_23	*RferOBP23*	OBP11 ANE37555
RPW1_contig_23127	not included*	
RPW1_contig_77	RferOBP77	
RPW1_contig_107	*RferOBP107*	OBP10 ANE37554
RPW1_contig_382	*RferOBP382*	
RPW1_contig_446	*RferOBP446*	OBP7 ANE37551
RPW1_contig_1768	*RferOBP1768*	OBP4 ANE37548
RPW1_contig_3937	*RferOBP3937*	
RPW1_contig_3997	*RferOBP3997*	
RPW1_contig_4661	*RferOBP4661*	
RPW1_contig_11442	not included*	
RPW1_contig_12010	*RferOBP12010*	
RPW1_contig_14025	*RferOBP14025*	
RPW1_contig_23691	*RferOBP23691*	
RPW1_contig_28119	*RferOBP28119*	
RPW1_contig_29381	*RferOBP29381*	
RPW1_contig_33721	*RferOBP33721*	
RPW1_contig_10788	*RferOBP10788*	
RPW1_contig_12481	*RferOBP12481*	
RPW1_contig_12511	*RferOBP12511*	
RPW1_contig_14511	*RferOBP14511*	
RPW1_contig_16551	*RferOBP16551*	
RPW1_contig_1689	*RferOBP1689*	OBP 5 & 6 (ANE37549 & ANE37550)
RPW1_contig_17793	*RferOBP17793*	
RPW1_contig_19755	*RferOBP19755*	
RPW1_contig_2374	*RferOBP2374*	
RPW1_contig_257	*RferOBP257*	OBP 1 ANE37545
RPW1_contig_29	*RferOBP29*	
RPW1_contig_3199	*RferOBP3199*	
RPW1_contig_3213	*RferOBP3213*	
RPW1_contig_7073	*RferOBP7073*	
RPW1_contig_8586	*RferOBP8586*	
RPW1_contig_9136	*RferOBP9136*	
RPW1_contig_981	*RferOBP981*	
RPW1_contig_9915	*RferOBP9915*	
RPW_OBP_Unigene 1	*RferOBPu1*	OBP 2 ANE37546
RPW_OBP_Unigene 2	*RferOBPu2*	
RPW_OBP_Unigene 3	*RferOBPu3*	
RPW_contig_1917	not included**	OBP 9 ANE37553
RPW_contig_1253	not included**	OBP 3 ANE37547
RWP_contig_272	not included**	OBP 8 ANE37552

* low similarities found in the BLASTx

**higher expression in RPW whole body transcriptome
