# Supplementary material for: Silencing the Odorant Binding Protein RferOBP1768 Reduces the Strong Preference of Palm Weevil for the Major Aggregation Pheromone Compound Ferrugineol
Source: Front Physiol. 2018 Mar 21;9:252. doi: 10.3389/fphys.2018.00252 (PMC5871713; doi:10.3389/fphys.2018.00252)
Supplement: Supplementary file 3 [file Table3.pdf]

Table S3. The significance in olfactometer preferences exhibited by each group of insects were analyzed by one-way ANOVA followed by LSD method. *P* values are compared within groups and provided in the table (α-level of significance *P*<0.05).

		NI	NFW	*RferOBP23*	*RferOBP107*	*RferOBPu1*	*RferOBP1768*
Response to Pheromone	NI	0	.590	.000	.000	.000	.000
	NFW	.590	0	.000	.000	.000	.000
	RferOBP23	.000	.000	0	.000	.000	.047
	RferOBP107	.000	.000	.000	0	.856	.000
	RferOBPu1	.000	.000	.000	.856	0	.000
	RferOBP1768	.000	.000	.047	.000	.000	0
Response to Air	NI	0	.290	.000	.000	.000	.000
	NFW	.290	0	.000	.002	.001	.000
	RferOBP23	.000	.000	0	.017	.033	.006
	RferOBP107	.000	.002	.017	0	.719	.000
	RferOBPu1	.000	.001	.033	.719	0	.000
	RferOBP1768	.000	.000	.006	.000	.000	0
No Response	NI	0	.165	.001	.030	.072	.005
	NFW	.165	0	.000	.002	.005	.000
	RferOBP23	.001	.001	0	.072	.030	.343
	RferOBP107	.030	.030	.072	0	.631	.343
	RferOBPu1	.072	.005	.030	.631	0	.165
	RferOBP1768	.005	.000	.343	.343	.165	0
