# Supplementary material for: Silencing the Odorant Binding Protein RferOBP1768 Reduces the Strong Preference of Palm Weevil for the Major Aggregation Pheromone Compound Ferrugineol
Source: Front Physiol. 2018 Mar 21;9:252. doi: 10.3389/fphys.2018.00252 (PMC5871713; doi:10.3389/fphys.2018.00252)
Supplement: Supplementary file 4 [file Table4.pdf]

Table S4. The significance in EAG responses exhibited by each group of insects were analyzed by Turkey's HSD method, and *P* values are compared within groups and provided in the table (α- level of significance *P*<0.05). Values with significant differences were highlighted in bold.

		NI	*RferOBP23*	*RferOBP107*	*RferOBPu1*	*RferOBP1768*
Phe1	NI	0	.589	.885	1.000	**.015**
	RferOBP23	.589	0	.190	.637	.153
	RferOBP107	.885	.190	0	.850	**.004**
	RferOBPu1	1.000	.637	.850	0	**.018**
	RferOBP1768	**.015**	.153	**.004**	**.018**	0
Phe2	NI	0	.951	1.000	.357	.888
	RferOBP23	.951	0	.927	.731	.524
	RferOBP107	1.000	.927	0	.318	.920
	RferOBPu1	.357	.731	.318	0	.099
	RferOBP1768	.888	.524	.920	.099	0
Ethyl Acetate	NI	0	.394	.688	.735	**.015**
	RferOBP23	.394	0	.980	.966	.252
	RferOBP107	.688	.980	0	1.000	.114
	RferOBPu1	.735	.966	1.000	0	.100
	RferOBP1768	**.015**	.252	.114	.100	0
