# Supplementary material for: Silencing the Odorant Binding Protein RferOBP1768 Reduces the Strong Preference of Palm Weevil for the Major Aggregation Pheromone Compound Ferrugineol
Source: Front Physiol. 2018 Mar 21;9:252. doi: 10.3389/fphys.2018.00252 (PMC5871713; doi:10.3389/fphys.2018.00252)
Supplement: Supplementary file 5 [file Table5.pdf]

Table S5. EAG responses of non-injected and dsRNA injected insects towards Phe1, Phe2 and Ethyl Acetate were provided as amplitude in mV. All EAG recordings were taken at 20mV with 20 s intervals. SEM is provided in the parenthesis. Homogenous subsets within the groups were identified using Waller-Duncan method ($\alpha = 0.05$) and represented as 'a', 'b' or 'a,b' with significance level measured using one-way ANOVA at $P<0.05$.

	Phe1	Phe2	Ethyl Acetate
NI	8.12 (0.24)[b]	4.26 (0.24)[a]	5.50 (0.24)[b]
RferOBP1768	3.09 (0.49)[a]	3.52 (0.49)[a]	2.26 (0.49)[a]
RferOBP23	5.44 (1.02)[a,b]	4.50 (0.94)[a]	3.69 (0.64)[a,b]
RferOBP107	9.26 (1.53)[b]	3.69 (0.80)[a]	4.45 (0.80)[a,b]
RferOBPu1	8.01 (0.74)[b]	6.64 (1.03)[a]	4.52 (0.27)[a,b]
F value	7.517	2.276	4.450
df value	4	4	4
P value	*0.005*	*0.133*	*0.025*
