# Supplementary material for: Silencing the Odorant Binding Protein RferOBP1768 Reduces the Strong Preference of Palm Weevil for the Major Aggregation Pheromone Compound Ferrugineol
Source: Front Physiol. 2018 Mar 21;9:252. doi: 10.3389/fphys.2018.00252 (PMC5871713; doi:10.3389/fphys.2018.00252)

**Figure S1:** Complete gel images provided as a supplementary material for the OBP tissue specific expression study results (Figure 1). The tissues in wells are represented in the order as AM (male antenna), AF (female antenna), Sn (male snout), Lg (male legs), Thx (male thorax), Ab (male abdomen) and Wg (male wings).

| No. | OBP name | AM AF Sn Lg Thx Ab Wg |
|---|---|---|
| 1 | *RferOBPu1* | 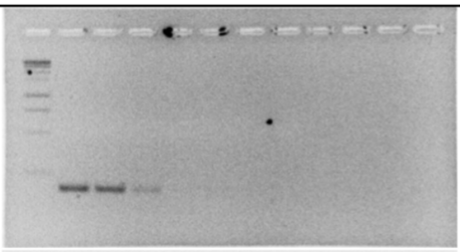 |
| 2 | *RferOBPu2* | 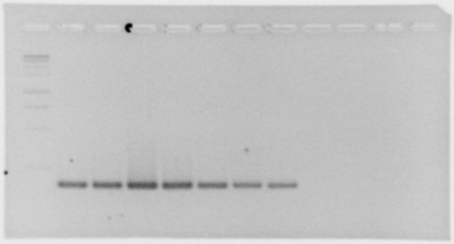 |
| 3 | *RferOBPu3* | 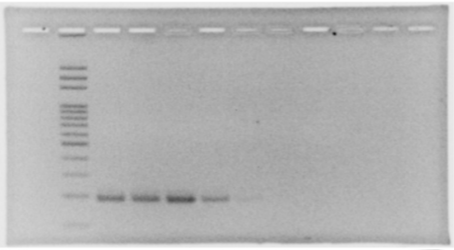 |
| 4 | *RferOBP2374* | 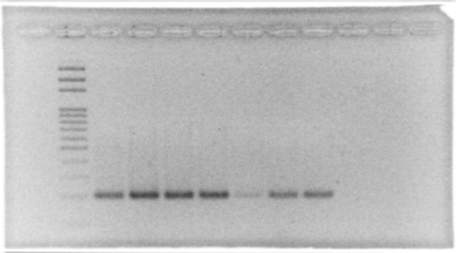 |
| 5 | *RferOBP257* | 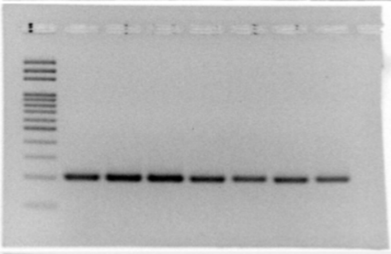 |
| 6 | *RferOBP29* | 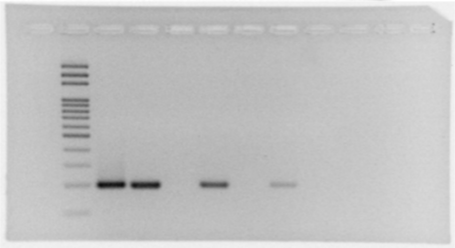 |

7 *RferOBP3199*

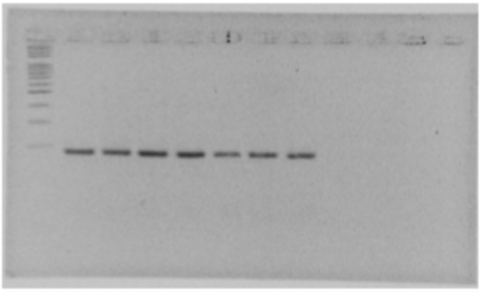

8 *RferOBP3213*

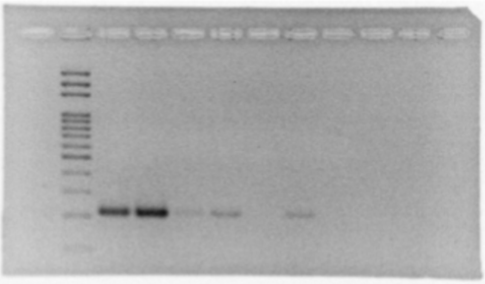

9 *RferOBP7073*

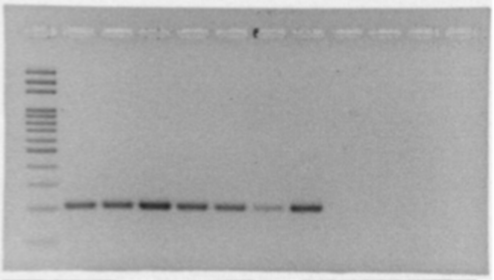

10 *RferOBP8586*

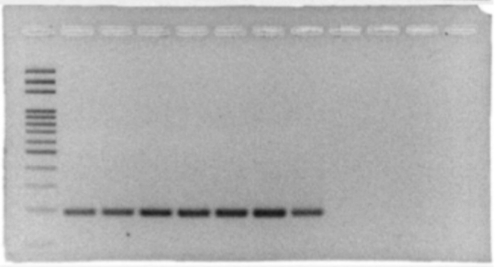

11 *RferOBP9136*

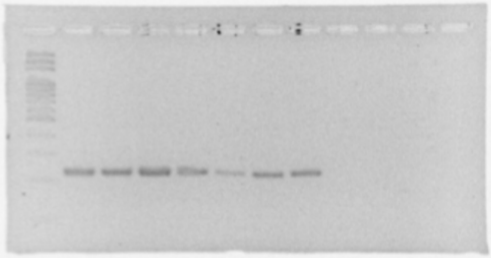

12 *RferOBP981*

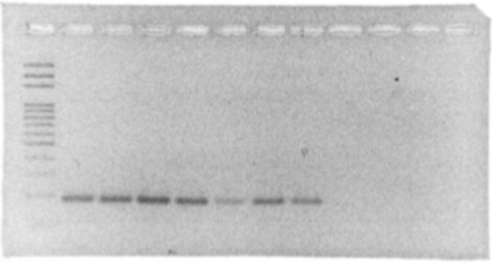

13 *RferOBP9915* Not amplified

14 *RferOBP77*

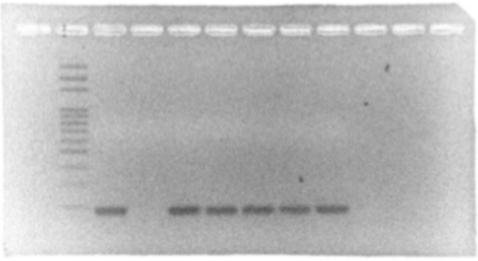

15 *RferOBP382*

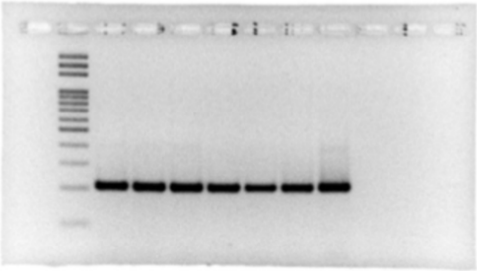

16 *RferOBP446*

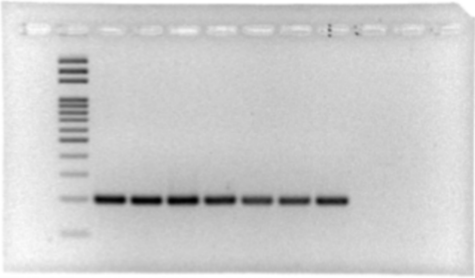

17 *RferOBP1768*

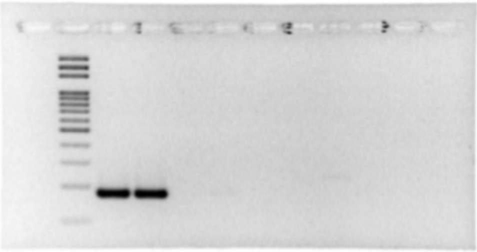

18 *RferOBP3937*

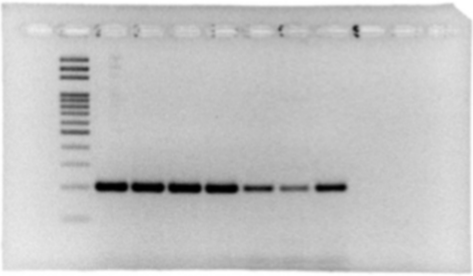

19 *RferOBP3997*

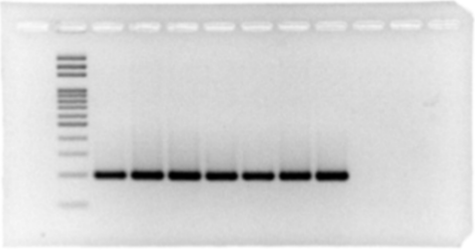

20 *RferOBP4661*

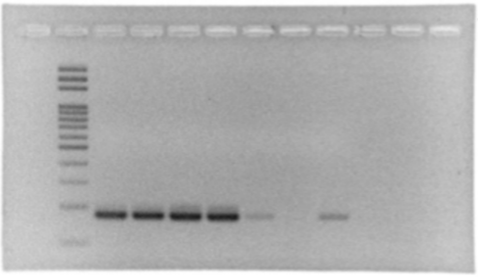

21 *RferOBP010*

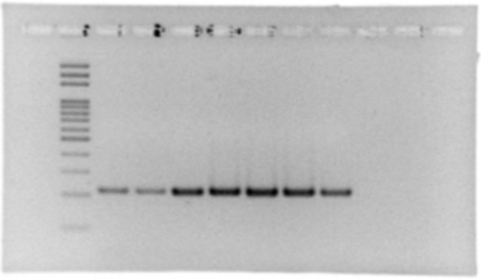

22 *RferOBP14025*

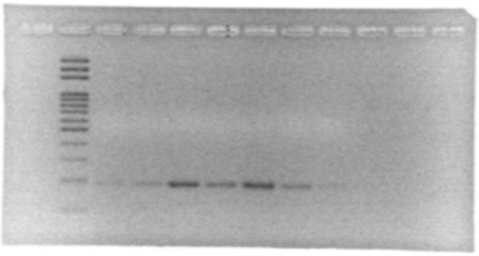

23 *RferOBP3691*

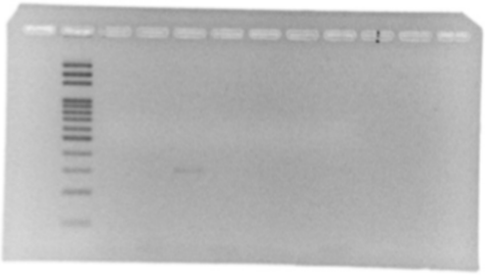

24 *RferOBP8119*

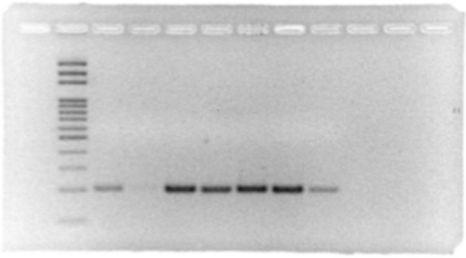

25 *RferOBP9381*

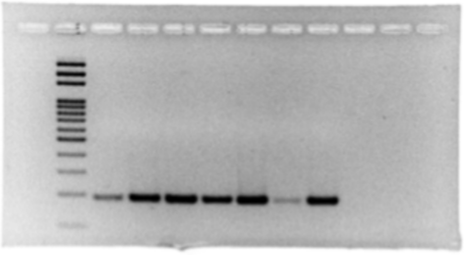

26 *RferOBP33721*

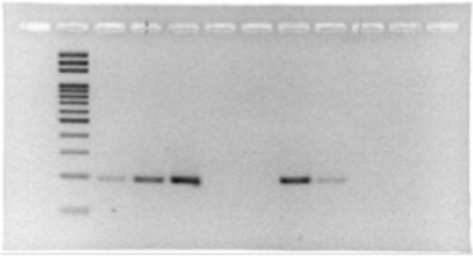

27 *RferOBP10788*

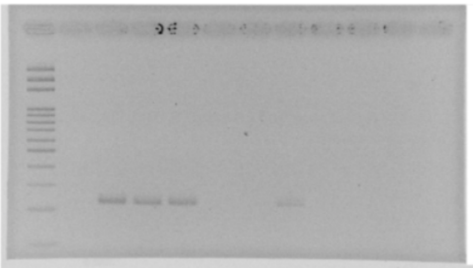

28 *RferOBP12481*

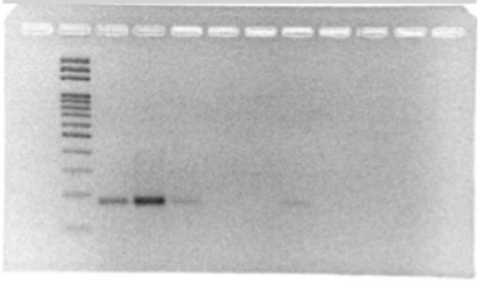

29 *RferOBP12511*

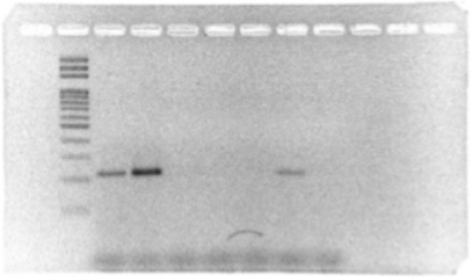

30 *RferOBP14551*

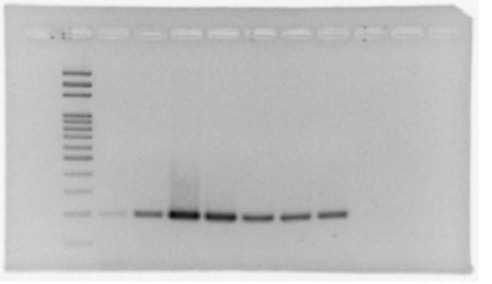

31 *RferOBP16551*

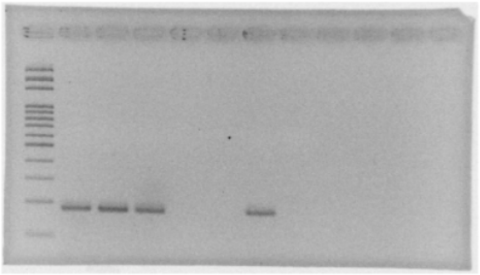

32 *RferOBP1689*

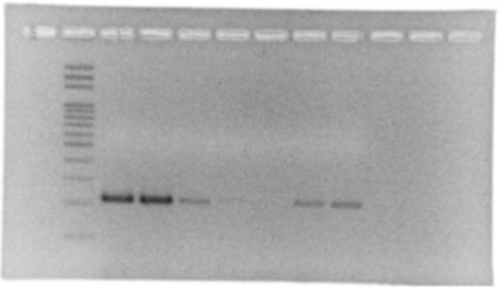

33 *RferOBP17793*

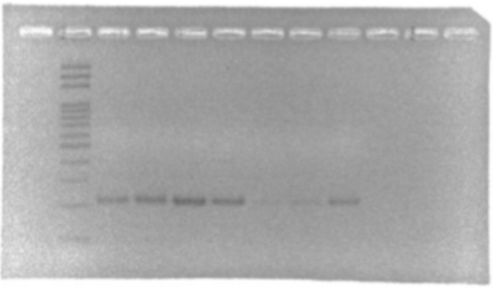

34 *RferOBP19755*

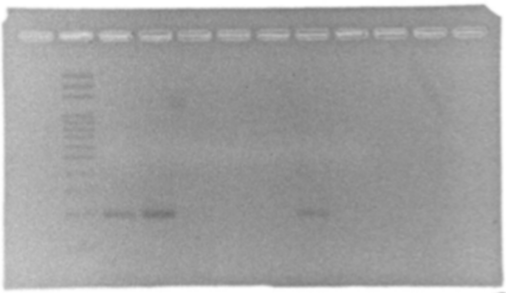

35 *RferOBP23*

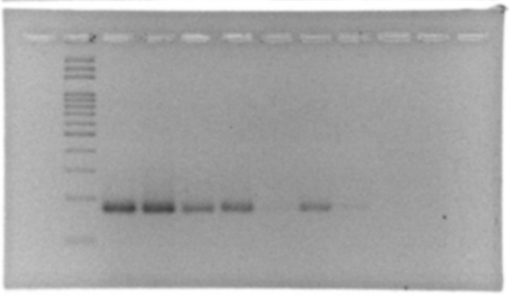

36 *RferOBP107*

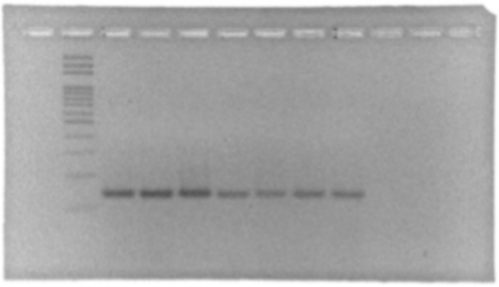

*RferTubulin*

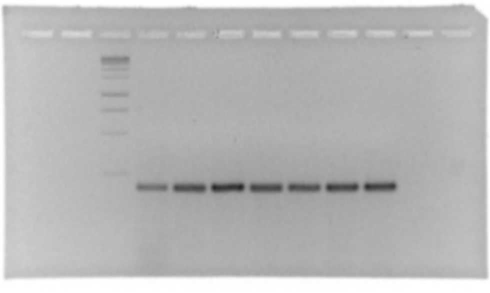

Supplement: Supplementary file 6 [file Image1.PDF]
