# Supplementary material for: Silencing the Odorant Binding Protein RferOBP1768 Reduces the Strong Preference of Palm Weevil for the Major Aggregation Pheromone Compound Ferrugineol
Source: Front Physiol. 2018 Mar 21;9:252. doi: 10.3389/fphys.2018.00252 (PMC5871713; doi:10.3389/fphys.2018.00252)
Supplement: Supplementary file 7 [file Image2.PDF]

Figure S2. A neighbour-joining (NJ) rooted tree of annotated OBPs for *R. ferrugineus* (Rfer), *Bombyx mori* (Bmori), *Anomala osakana* (Aosa), *A. octiescosrara* (Aoct), *A. cuprea* (Acup), *P. japonica* (Pjap), *Drosophila* and *Tribolium castaneum* (Tcas) OBPs. The tree was constructed by the neighbour-joining method using MEGA6 [statistical method: NJ; phylogeny test: bootstrap method; model: JTT model and gaps/missing data treatment: pairwise deletion] and generated with a bootstrap procedure using 1000 replications. The branches are coloured, and bootstrap values above 50% are indicated. *Bombyx mori* OBPs (Gong et al. 2009) were used as a reference to classify *R. ferrugineus* OBPs. Different colour codes indicate OBP subfamilies. Red: Minus-C, green: CRLBP, black: ABPI, pink: Plus-C, magenta: PBP/GOBP and blue: ABPII. The *RferOBP1768* and *RferOBP23* clades are highlighted in yellow and green, respectively. Sequence logos of the aligned *R. ferrugineus RferOBP1768* and *RferOBP23* orthologues are given in Figure S3. The protein sequences were aligned using MUSCLE. The branch containing *Drosophila* OBP LUSH (PDB: 2GTE) was used as an outgroup to root the tree. Phylogenetic tree was visualized with the software FigTree (http://tree.bio.ed.ac.uk/software/figtree/). Scale: 0.4 amino acid substitutions per site.

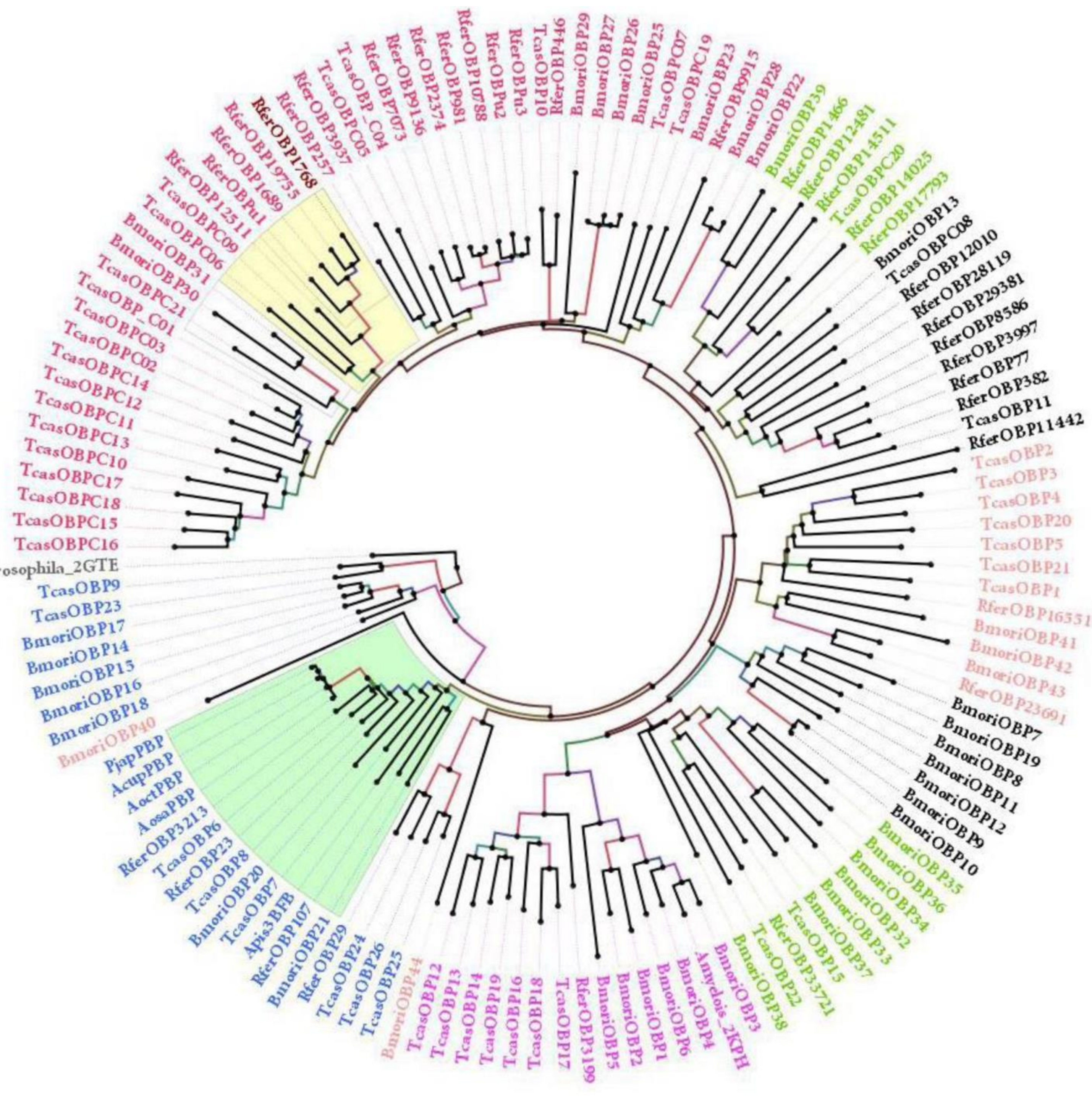

Drosophila_2GTE
TcasOBPC16
TcasOBPC15
TcasOBPC18
TcasOBPC17
TcasOBPC10
TcasOBPC13
TcasOBPC11
TcasOBPC12
TcasOBPC14
TcasOBPC02
TcasOBPC03
TcasOBP_C01
TcasOBPC21
BmoriOBP30
BmoriOBP31
BmoriOBPC06
TcasOBPC09
RferOBP12511
RferOBPu1
RferOBP1689
RferOBP19755
RferOBP1768
RferOBP257
RferOBP3937
TcasOBPC05
TcasOBP_C04
RferOBP7073
RferOBP9136
RferOBP2374
RferOBP981
RferOBP10788
RferOBPu2
RferOBPu3
TcasOBP10
RferOBP446
BmoriOBP29
BmoriOBP27
BmoriOBP26
BmoriOBP25
TcasOBPC07
TcasOBPC19
BmoriOBP23
RferOBP9915
BmoriOBP28
BmoriOBP22
BmoriOBP39
RferOBP1466
RferOBP12481
RferOBP14511
TcasOBPC20
RferOBP14025
RferOBP17793
BmoriOBP13
TcasOBPC08
RferOBP12010
RferOBP28119
RferOBP29381
RferOBP8586
RferOBP3997
RferOBP77
RferOBP382
TcasOBP11
RferOBP11442
TcasOBP2
TcasOBP3
TcasOBP4
TcasOBP20
TcasOBP5
TcasOBP21
TcasOBP1
RferOBP16351
BmoriOBP41
BmoriOBP42
BmoriOBP43
RferOBP23691
BmoriOBP7
BmoriOBP19
BmoriOBP8
BmoriOBP11
BmoriOBP12
BmoriOBP9
BmoriOBP10
BmoriOBP35
BmoriOBP36
BmoriOBP34
BmoriOBP32
BmoriOBP33
BmoriOBP37
TcasOBP15
RferOBP33721
TcasOBP22
BmoriOBP38
BmoriOBP3
Amyelois_2KPH
BmoriOBP4
BmoriOBP6
BmoriOBP1
BmoriOBP2
BmoriOBP5
RferOBP3199
TcasOBP17
TcasOBP18
TcasOBP16
TcasOBP19
TcasOBP14
TcasOBP13
TcasOBP12
BmoriOBP44
TcasOBP25
TcasOBP26
TcasOBP24
RferOBP29
BmoriOBP21
RferOBP107
Apis3BFB
TcasOBP7
BmoriOBP20
TcasOBP8
RferOBP23
TcasOBP6
RferOBP3213
AosaPBP
AoctPBP
AcupPBP
PjapPBP
BmoriOBP40
BmoriOBP18
BmoriOBP16
BmoriOBP15
BmoriOBP14
BmoriOBP17
TcasOBP23
TcasOBP9
