# Supplementary material for: Silencing the Odorant Binding Protein RferOBP1768 Reduces the Strong Preference of Palm Weevil for the Major Aggregation Pheromone Compound Ferrugineol
Source: Front Physiol. 2018 Mar 21;9:252. doi: 10.3389/fphys.2018.00252 (PMC5871713; doi:10.3389/fphys.2018.00252)

Figure S3. Sequence logo of the aligned *R. ferrugineus RferOBP1768* clade and *RferOBP23* clade (refer Figure S2). The logo displays the frequencies of amino acids at each position, with the height of a letter reflecting the raw residue frequencies. Amino acids are colored according to classic properties (NA) (refer: http://weblogo.threeplusone.com/create.cgi). The logo was created using WebLogo 3.1.

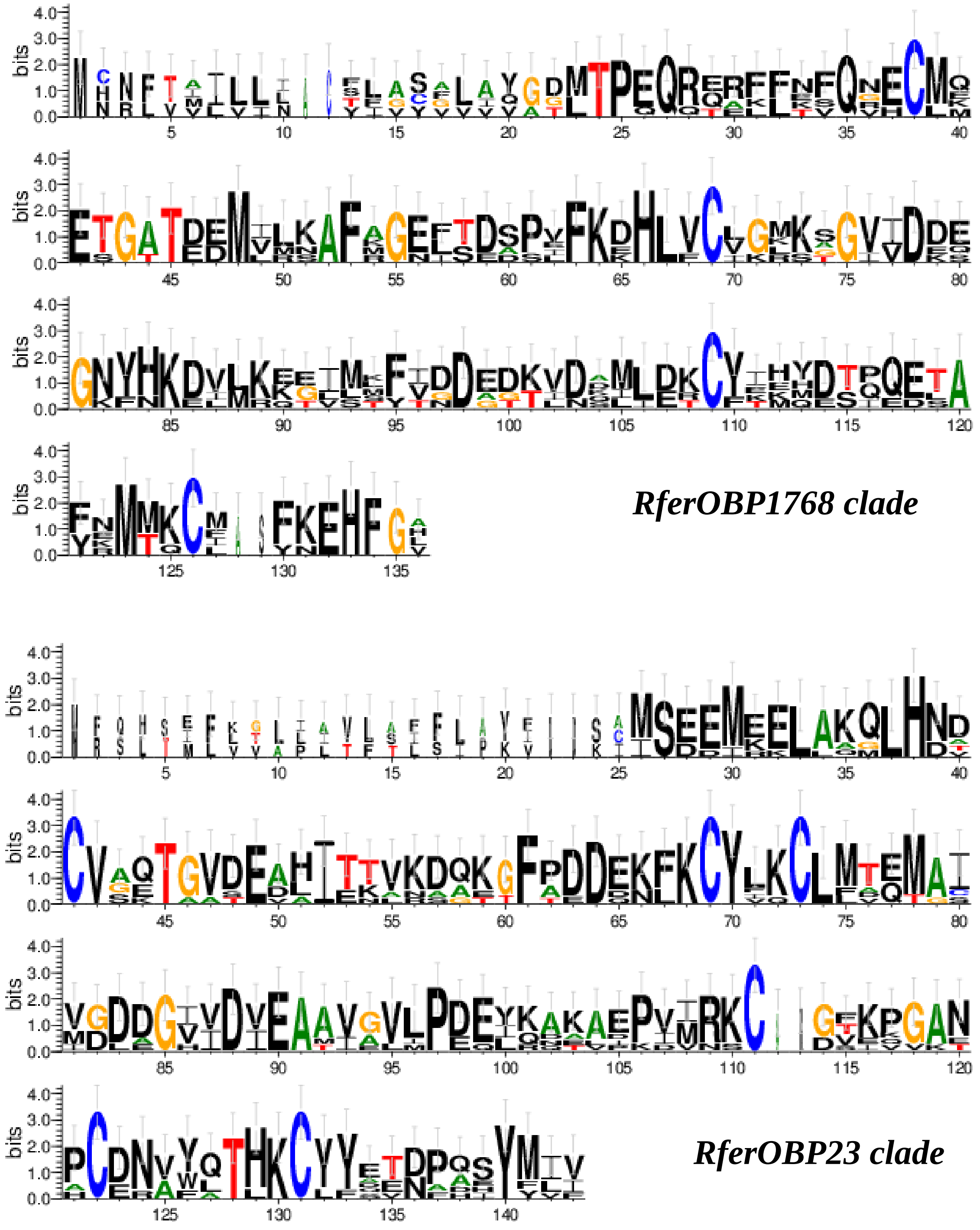

Supplement: Supplementary file 8 [file Image3.PDF]
