# Supplementary material for: Silencing the Odorant Binding Protein RferOBP1768 Reduces the Strong Preference of Palm Weevil for the Major Aggregation Pheromone Compound Ferrugineol
Source: Front Physiol. 2018 Mar 21;9:252. doi: 10.3389/fphys.2018.00252 (PMC5871713; doi:10.3389/fphys.2018.00252)

**Figure S4**. Three-dimensional structures in ribbon representation of *RferOBP23* prepared based on the highly ranked structural homologue, insect pheromone/odorant binding proteins OBP1 (PDB: 3BJH) using PHYRE tool. Structures have been visualized using PYMOL *v*2.0.4. Structures have been visualized using PYMOL *v*2.0.4. The rainbow colouring mode is applied to the Cα ribbons: the N-terminus (Nt) is blue, and the C-terminus (Ct) is red. The six helices (α1-α6) are indicated. The ligand (Phe1: 4RS,5RS)-4-methylnonan-5-ol) is represented as spheres. The predicted binding site for *RferOBP23* corresponded to Ile79 and Asp80, which are likely involved in pheromone binding.

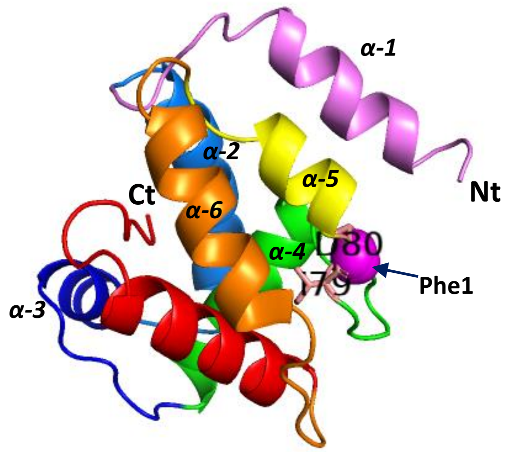

Supplement: Supplementary file 9 [file Image4.PDF]
